# Using native and synthetic genes to disrupt inositol pyrophosphates and phosphate accumulation in plants

**DOI:** 10.1093/plphys/kiae582

**Published:** 2024-10-30

**Authors:** Catherine Freed, Branch Craige, Janet Donahue, Caitlin Cridland, Sarah Phoebe Williams, Chris Pereira, Jiwoo Kim, Hannah Blice, James Owen, Glenda Gillaspy

**Affiliations:** Department of Biochemistry, University of Wisconsin-Madison, Madison, WI 53706, USA; Department of Biochemistry, Virginia Tech, Blacksburg, VA 24061, USA; Department of Biochemistry, Virginia Tech, Blacksburg, VA 24061, USA; Department of Biochemistry, Virginia Tech, Blacksburg, VA 24061, USA; Department of Biology, The College of William & Mary, Williamsburg, VA 23185, USA; Department of Biochemistry, Virginia Tech, Blacksburg, VA 24061, USA; Department of BioSciences, Rice University, Houston, TX 77005, USA; Application Technology Research Unit, US Department of Agriculture, Agricultural Research Service, Wooster, OH 44691, USA; Application Technology Research Unit, US Department of Agriculture, Agricultural Research Service, Wooster, OH 44691, USA; Department of Biochemistry, University of Wisconsin-Madison, Madison, WI 53706, USA

## Abstract

Inositol pyrophosphates are eukaryotic signaling molecules that have been recently identified as key regulators of plant phosphate sensing and homeostasis. Given the importance of phosphate to current and future agronomic practices, we sought to design plants, which could be used to sequester phosphate, as a step in a phytoremediation strategy. To achieve this, we expressed diadenosine and diphosphoinositol polyphosphate phosphohydrolase (DDP1), a yeast (*Saccharomyces cerevisiae*) enzyme demonstrated to hydrolyze inositol pyrophosphates, in *Arabidopsis thaliana* and pennycress (*Thlaspi arvense*), a spring annual cover crop with emerging importance as a biofuel crop. DDP1 expression in *Arabidopsis* decreased inositol pyrophosphates, activated phosphate starvation response marker genes, and increased phosphate accumulation. These changes corresponded with alterations in plant growth and sensitivity to exogenously applied phosphate. Pennycress plants expressing DDP1 displayed increases in phosphate accumulation, suggesting that these plants could potentially serve to reclaim phosphate from phosphate-polluted soils. We also identified a native *Arabidopsis* gene, *Nucleoside diphosphate-linked moiety X 13* (*NUDIX13*), which we show encodes an enzyme homologous to DDP1 with similar substrate specificity. *Arabidopsis* transgenics overexpressing NUDIX13 had lower inositol pyrophosphate levels and displayed phenotypes similar to DDP1-overexpressing transgenics, while *nudix13-1* mutants had increased levels of inositol pyrophosphates. Taken together, our data demonstrate that DDP1 and NUDIX13 can be used in strategies to regulate plant inositol pyrophosphates and could serve as potential targets for engineering plants to reclaim phosphate from polluted environments.

## Teaser

Hydrolyzing inositol pyrophosphates increases phosphate accumulation in plants and presents a proof of concept for engineering plants to reclaim phosphate from polluted environments.

## Introduction

Inorganic phosphate (P*i*), a required nutrient for genetic maintenance, cellular function, and energy metabolism, is arguably the greatest plant growth-limiting macronutrient and is indispensable for food production and security on a global scale (Vance et al. 2003). While critically important, P*i* is scarce in many agricultural soils and is a rapidly depleting, nonrenewable resource (Cordell et al. 2009). While phosphate deficiency has great global agricultural implications, the overuse of P*i*-rich manure and chemical fertilizers to fields often leads to excessive P*i* accumulation in soil (Menezes-Blackburn et al. 2018). Moreover, P*i* runoff from farmland and urban areas into the watersheds can lead to harmful algal blooms (Gobler 2020). The combination of impending P*i* shortages, fertilizer overusage and the negative toxic environmental impacts, described as the P*i* crisis, will only become further aggravated with climate change as well as a lack of policy and public awareness (Vaccari 2009; Cordell and White 2011). In the face of these challenges, it is crucial to understand how plants are able to utilize P*i* in their environment to develop new solutions to combat the P*i* crisis. Under depleted P*i* conditions, plants employ molecular mechanisms, collectively known as the P*i* starvation response (PSR), to reprioritize growth patterns to increase P*i* uptake and redistribute P*i* from existing cells (Rouached et al. 2010; Cho et al. 2021; Wang et al. 2021; Madison et al. 2023). The PSR is modulated by complex signaling networks, and while its regulation is not completely understood, emerging evidence strongly supports an integral role for inositol pyrophosphates (PP-InsPs) as central regulators of the PSR (Wild et al. 2016; Dong et al. 2019; Zhu et al. 2019).

PP-InsPs and their precursors, inositol phosphates (InsPs), are important messengers across eukaryotes (Tsui and York 2010; Thota and Bhandari 2015; Azevedo and Saiardi 2017; Shears 2018; Morrissette and Rolfes 2020; Laha et al. 2021). PP-InsPs and InsPs consist of a 6-carbon *myo*-inositol ring and are sequentially phosphorylated by evolutionarily conserved enzymes (Irvine and Schell 2001; Williams et al. 2015; Livermore et al. 2016; Shears 2018). The number and position of P*i* moieties on the ring convey different intracellular messages (Irvine and Schell 2001; Shears 2015; Livermore et al. 2016). InsP_6_, also known as phytate when chelated with metals, is the most abundant InsP species found in plants and is important for P*i* sensing and storage (Raboy et al. 2001). InsP_6_ is implicated as a structural component in plant auxin signaling through binding with the auxin receptor, transport inhibitor 1 (Tan et al. 2007). In plants, InsP_6_ can be acted on sequentially by 2 enzymes, the inositol 3,4,5,6-tetrakisphosphate 1-kinase (ITPK), and the diphosphoinositol pentakisphosphate 1-kinase (named VIP or VIH), to form the 2 known PP-InsPs, commonly referred to as InsP_7_ and InsP_8_ (Adepoju et al. 2019; Laha et al. 2019; Whitfield et al. 2020). InsP_7_ and InsP_8_ are hypothesized to bind and regulate the jasmonate receptor CORONATINE INSENSITIVE1 (COI-1) (Laha et al. 2015, 2016) although InsP_5_ has also been implicated due to its presence within the crystallized COI-1 complex (Sheard et al. 2010). Understanding the specific roles of PP-InsPs in these and other plant signaling pathways is an area of emerging interest and requires further examination.

**Figure 1.**
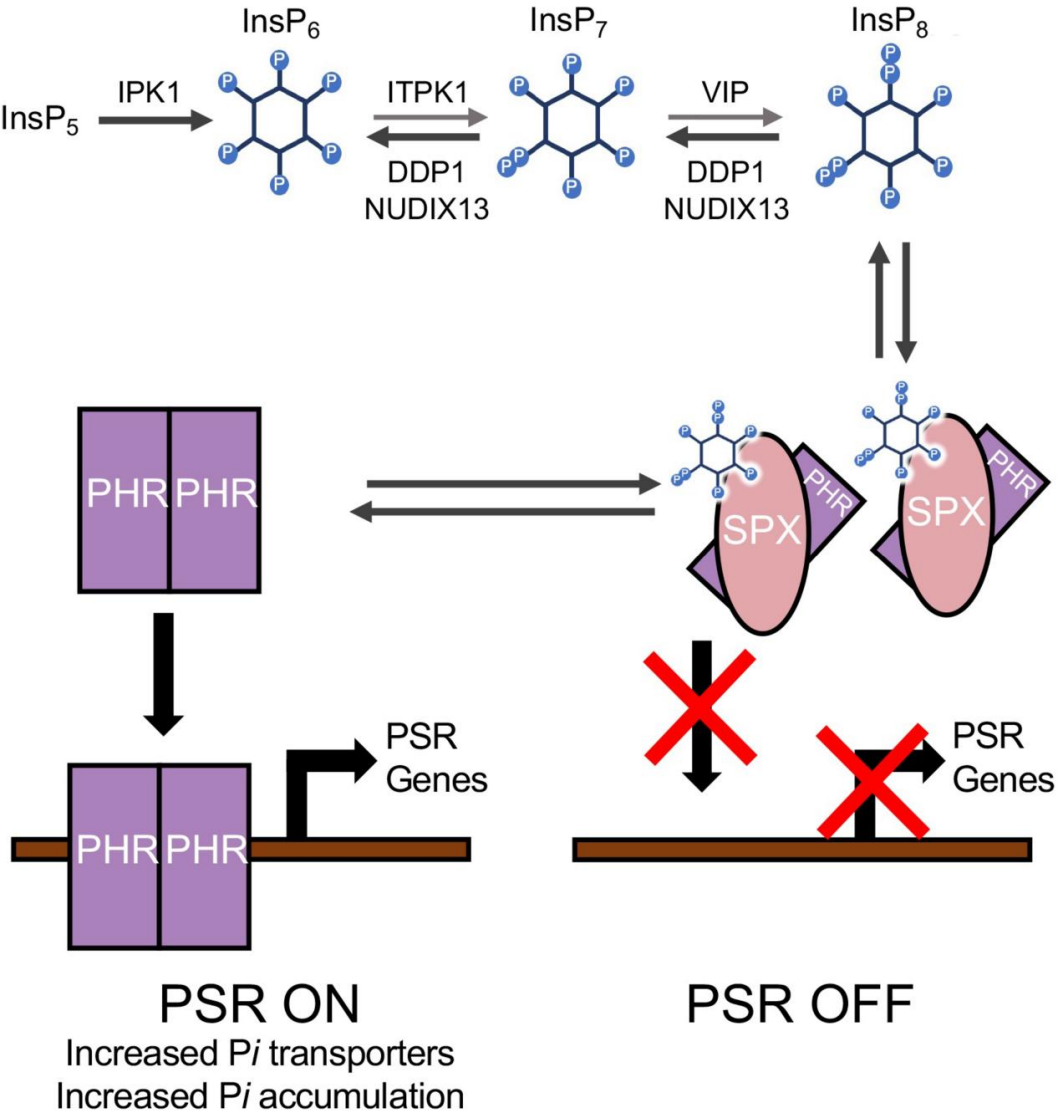
Simplified model for PP-InsP synthesis, degradation, and regulation of PSR genes. IPK1 synthesizes InsP_6_, ITPK enzymes synthesize InsP_7_, and VIP enzymes synthesize InsP_8_ in *Arabidopsis*. Transgenic expression of DDP1 (*S. cerevisiae*) or NUDIX13 (*Arabidopsis*) is predicted to hydrolyze InsP_7_ and InsP_8_ in planta. It is important to note that this is a simplified figure and does not address specific InsP isomers or the newly discovered isomers of InsP_7_ (Riemer et al. 2021; Laha et al. 2022). Under P*i*-deplete conditions, dimerized PHR binds to the P1BS promoter and upregulates PSR genes (“PSR ON”) (Zhou et al. 2021). Under P*i*-replete conditions, InsP_8_ is assumed to be the controller to turn the PSR off. InsP_8_ binds to SPX and this SPX:InsP_8_ complex disrupts the PHR dimer and inhibits PHR-mediated transcription of PSR genes.

The role of PP-InsPs as critical players in eukaryotic P*i* sensing has been facilitated by the examination of loss-of-function mutants in the InsP and PP-InsP synthesis pathways (Dong et al. 2019; Zhu et al. 2019; Land et al. 2021). Specifically, depleting InsP_8_ in planta leads to alterations in a plant's ability to properly sense and respond to P*i* (Fig. 1) (Dong et al. 2019; Zhu et al. 2019; Land et al. 2021). *Arabidopsis* loss-of-function double mutants for both InsP_8_ synthetic enzymes (VIP1 and VIP2, which are also referred to as VIH2 and VIH1, respectively) have depleted levels of PP-InsPs, as well as altered physiological responses to P*i*, which include increased P*i* accumulation, PSR gene induction, and impacted growth (Dong et al. 2019; Zhu et al. 2019). These data support a model for control of the PSR transcriptional response where InsP_8_ controls association of the Phosphate Starvation Response Regulator 1 (PHR1) transcription factor with its binding partner, SPX1 (named after *Saccharomyces cerevisiae* proteins Syg1 and Pho81 and mammalian protein Xpr1; Puga et al. 2014) preventing PHR1-mediated induction of PSR genes (Fig. 1). The SPX domain present within SPX1 has been shown via binding assays to bind InsP_8_ at a high affinity (Wild et al. 2016; Ried et al. 2021). A recent study shows that a rice SPX2/InsP_6_ (serving as a substitute for InsP_8_)/PHR2 complex inhibited the binding of PHR2 to DNA by disrupting PHR2 dimerization (Guan et al. 2022). Taken together, this suggests that InsP_8_ can be viewed as a proxy for intracellular P*i* levels and is likely the main molecule regulating PHR1-SPX complexes.

It is crucial to understand the extent to which InsPs and PP-InsPs contribute to plant P*i* homeostasis and to utilize this information to inform future translational approaches to address the global P*i* crisis. While *vih1-2 vih2-4* mutants directly target the enzymes that synthesize InsP_8_ (Zhu et al. 2019), the severe growth phenotypes of these mutants make it difficult to explore P*i* homeostasis in agronomic conditions. Furthermore, while previous studies focused on plant PP-InsP synthesis enzymes, the enzymes involved in plant PP-InsP degradation are much less characterized. Given this, we have decided to explore the utility PP-InsP phosphatases in impacting plant PP-InsP signaling. *Nu*cleoside *di*phosphate-linked moiety *X* (NUDIX) enzymes, along with the phosphatase domain of VIP enzymes (Mulugu et al. 2007; Shears et al. 2017) and SIW14/DSP-PFA enzymes (Aceti et al. 2008; Wang et al. 2018; Gaugler et al. 2022), are the most likely known PP-InsP degrading enzymes that could function to regulate cellular levels of PP-InsPs in plants. The NUDIX clan contains a highly diverse set of phosphohydrolases that are found in all domains of life (Srouji et al. 2017). A total of 27 NUDIX enzymes have been identified in *Arabidopsis* (Kraszewska 2008; Ogawa et al. 2008) and they share a similar, highly conserved NUDIX hydrolase motif similar to diadenosine and diphosphoinositol polyphosphate phosphohydrolase (DDP1) (Sheikh et al. 1998; Xu et al. 2002; Kraszewska 2008). Of these 27 enzymes, only the structures of NUDIX1 (Liu et al. 2018) and NUDIX7 (Tang et al. 2015) have been resolved. Furthermore, the enzymatic activities of about half of these *Arabidopsis* NUDIX enzymes have been empirically explored but the roles of many of the others remain unelucidated (Kraszewska 2008). Of what has been explored regarding the plant NUDIX enzymes, the role of these enzymes in PP-InsP signaling in plants lacks clarity.

We have focused on developing a synthetic biology approach to directly target and break down PP-InsPs in plants. For this study, we selected DDP1 (gene YOR163w), a phosphatase from *S. cerevisiae* containing a NUDIX hydrolase motif that hydrolyzes phosphoanhydride bonds from PP-InsPs, polyphosphates (polyP; polyP_n_), and diadenosine polyphosphates (Ap_n_A; specifically, Ap_5_A and Ap_6_A) (Safrany et al. 1999; Gunawardana et al. 2009; Lonetti et al. 2011; Kilari et al. 2013; Márquez-Moñino et al. 2021). Of these 3 molecule classes, only PP-InsPs have been detected in plants (Desai et al. 2014; Laha et al. 2015) and have a known synthesis pathway (Desai et al. 2014; Laha et al. 2015; Adepoju et al. 2019; Laha et al. 2019). With regard to PP-InsPs, DDP1 has been shown to hydrolyze InsP_8_ and InsP_7_ isomers, 1-InsP_7_ and 5-InsP_7_, with the strongest preference for hydrolyzing 1-InsP_7_ (Kilari et al. 2013). Here, we report that DDP1 overexpression in *Arabidopsis* decreases PP-InsP accumulation, increases P*i* accumulation over the course of plant development, and activates PSR genes. To identify NUDIX enzymes involved in PP-InsP degradation in planta, we have selected NUDIX13 given a protein BLAST using DDP1 as a query identified NUDIX13 as a strong candidate (*e* value of 2e^−10^) (Altschul et al. 1997; Altschul et al. 2005) as well as its prediction to hydrolyze PP-InsPs (Kraszewska 2008), and its ability to hydrolyze Ap_5_A and Ap_6_A (Olejnik et al. 2007), which are known DDP1 substrates (Safrany et al. 1999; Lonetti et al. 2011; Kilari et al. 2013; Márquez-Moñino et al. 2021). We hypothesize that NUDIX13 could hydrolyze PP-InsPs and increase plant P*i* accumulation. We engineered NUDIX13 gain-of-function transgenics and found that they exhibited similar phenotypes to DDP1 transgenics through P*i* toxicity phenotypes, decreased PP-InsPs, and increased P*i* accumulation. Herein, we demonstrate that DDP1 and NUDIX13 hydrolyze a group of substrates including InsP_7_, InsP_8_, Ap_5_A, and polyP in vitro and localize to the same subcellular compartments: nucleus and cytosol. Additionally, we explored the translational utility of these changes and transferred the *DDP1* gene into a cover crop species, *Thlaspi arvense* (pennycress), and showed that this cover crop model system can increase P*i* accumulation from soil. Ultimately, these unique *Arabidopsis* and pennycress transgenics will be useful in understanding the role of PP-InsPs in P*i* sensing as well as in informing phytoremediation strategies.

## Results

### DDP1 overexpression in *Arabidopsis* negatively impacts growth and P*i* sensitivity

**Figure 2.**
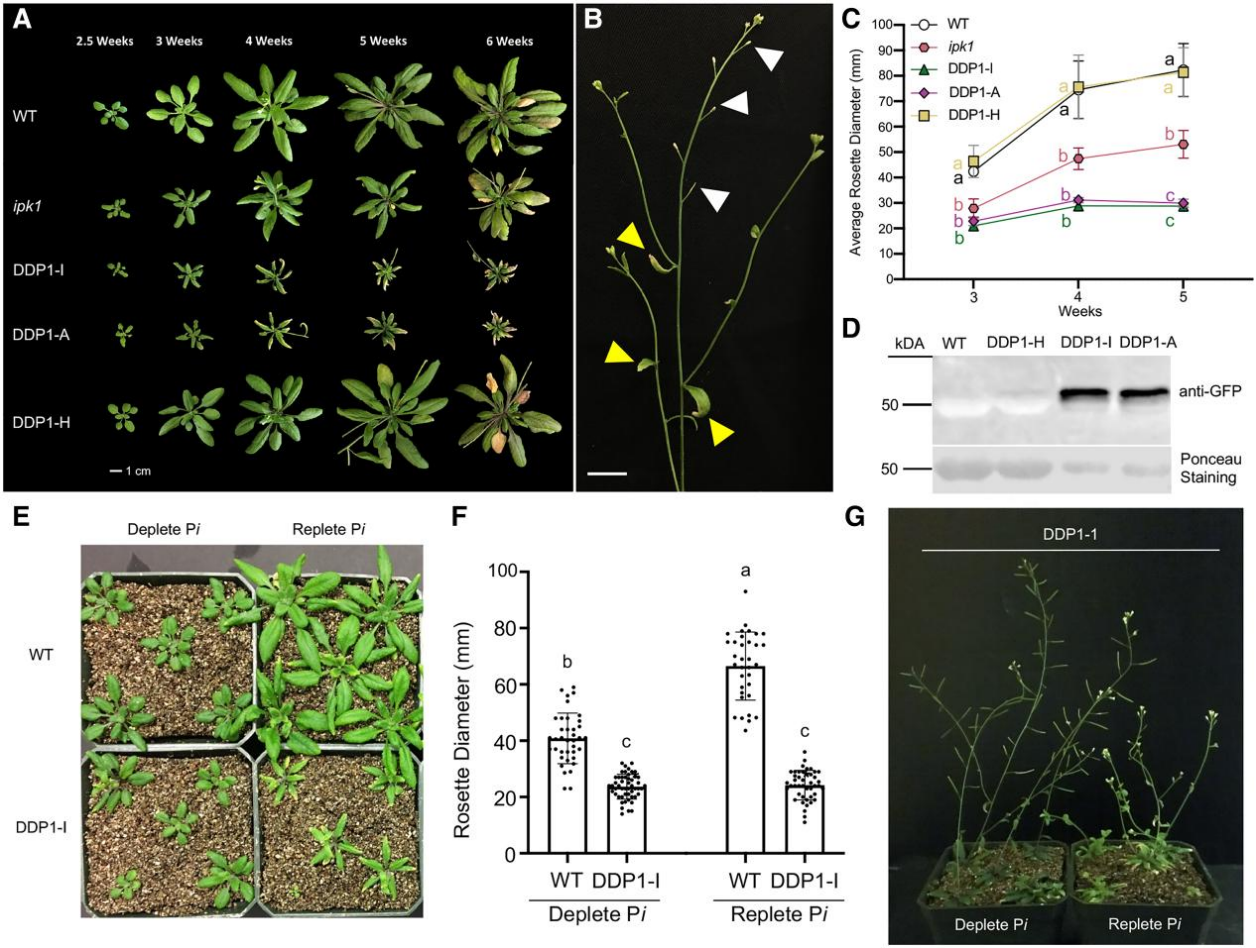
Characterization and comparison of DDP1 OX transgenics. **A)***Arabidopsis* rosette growth over the course of 6 wk, each image representative of *n* = 3 to 4 independent experiments containing 12 or more plants per genotype. Images were digitally extracted for comparison. Scale bar = 1 cm. **B)** Close-up view of aborting siliques in a 5-wk-old DDP1-I plant. The three white arrowheads at the top of the image indicate aborting siliques, and the three yellow arrowheads at the bottom of the image mark yellowing cauline leaves. Scale bar = 1 cm. **C)** Average rosette diameter over time. Each point represents *n* = 3 independent experiments with over 12 plants per genotype per experiment; error bars show Sd. Different letters indicate statistically significant means (Tukey honestly significant difference (HSD) test, *α* = 0.05). **D)** Immunoblot of 4-wk-old leaf tissue from WT and selected DDP1 OX transgenics. Ponceau staining shows Rubisco accumulation in all plants as a positive control (∼50 to 56 kDa). **E)** WT and DDP1 OX transgenics grown on vermiculite containing 0.5× MS media with 10 *μ*m KH_2_PO_4_ (deplete P*i*) or 1 mm KH_2_PO_4_ (replete P*i*) after 35 d. DDP1-I plants did not accumulate lesions under deplete P*i* and resemble WT plants on deplete P*i*. **F)** Rosette diameter measurements of WT and DDP1-I grown under deplete and replete P*i*. Each point represents an individual plant measurement from *n* = 2 independent experiments; over 35 plants per genotype and condition; error bars show Sd. Different letters indicate statistically significant means (Tukey HSD, *α* = 0.05). **G)** DDP1-I transgenics after 50 d of growth on vermiculite. DDP1-I lines grown on deplete P*i* did not have aborted siliques.

To reduce PP-InsPs in planta, transgenic lines were generated by overexpressing *S. cerevisiae DDP1* (YOR163w) (Cartwright and McLennan 1999; Lonetti et al. 2011; Kilari et al. 2013) fused to a C-terminal GFP tag under control of the CaMV 35S promoter in *Arabidopsis* Col-0 plants. DDP1 overexpression lines (DDP1 OX) were confirmed by immunoblotting, which revealed correlation of DDP1-GFP expression with a severe growth phenotype (Fig. 2, A to D). For this study, we selected 3 DDP1 OX lines expressing abundant or very low amounts of DDP1-GFP. DDP1-A and DDP1-I transgenics displayed abundant DDP1-GFP expression and are severely impacted in their growth (Fig. 2, A to D). These plants have a significantly reduced rosette diameter, as well as leaf tip necrosis and leaf chlorosis, phenotypes that are evocative of plant P*i* toxicity (Fig. 2, A to C) (Aung et al. 2006; Hu et al. 2011). As DDP1-I and DDP1-A plants begin the reproductive phase, abortion of developing embryos can be seen (Fig. 2B), leading to low recovery of seeds. In contrast, the DDP1-H line has barely detectable levels of DDP1-GFP protein accumulation, does not differ in growth from wild-type (WT) plants, and does not have negatively impacted reproductive phenotypes (Fig. 2, A to D). We compared DDP1 OX lines with a well-characterized PP-InsP-depleted mutant, *ipk1*. *ipk1* harbors a partial loss-of-function mutation in *INOSITOL-PENTAKISPHOSPHATE 2-KINASE 1*, the only known enzyme in plants to synthesize InsP_6_ from InsP_5_ (Stevenson-Paulik et al. 2005). *ipk1* mutants are significantly smaller than WT plants, as seen by a reduced rosette diameter, which is a phenotype observed in previous studies (Stevenson-Paulik et al. 2005; Kuo et al. 2014, 2018). DDP1-I, DDP1-A, and, to a lesser extent, *ipk1* plants exhibit leaf tip necrosis and chlorosis (Fig. 2, A to C), suggesting a sensitivity to P*i*.

To test the P*i* sensitivity of DDP1 OX plants, we focused on the DDP1-I line and grew plants hydroponically with either 10 *μ*m or 1 mm KH_2_PO_4_ for up to 50 d to simulate P*i*-deplete and P*i*-replete conditions, respectively. As expected, the growth of WT plants is greatly compromised by P*i*-deplete conditions, with a significant reduction in shoot growth (Fig. 2, E and F). In contrast, we found that DDP1 OX transgenics were more comparable in size to WT plants when grown under P*i*-deplete conditions though DDP1 OX transgenics were significantly smaller than WT under both conditions (Fig. 2, E and F). Additionally, while DDP1-I leaves are lighter in color and form brown necrotic lesions in P*i*-replete conditions, growth under P*i*-deplete conditions results in improvement in growth as seen by a darker leaf color and absence of necrotic lesions (Fig. 2G). A qualitative assessment of seed production indicated that growth of DDP1-I plants under P*i*-deplete conditions resulted in a rescue of the embryo abortive phenotype, as seen by DDP1 OX plants with stems and siliques that resembled WT plants after 50 d of growth (Fig. 2G). Leaf and seed phenotypes associated with DDP1 overexpression can be rescued under depleted P*i* conditions, indicating that these phenotypes are P*i* dependent.

### DDP1 overexpression reduces seedling PP-InsP levels

**Figure 3.**
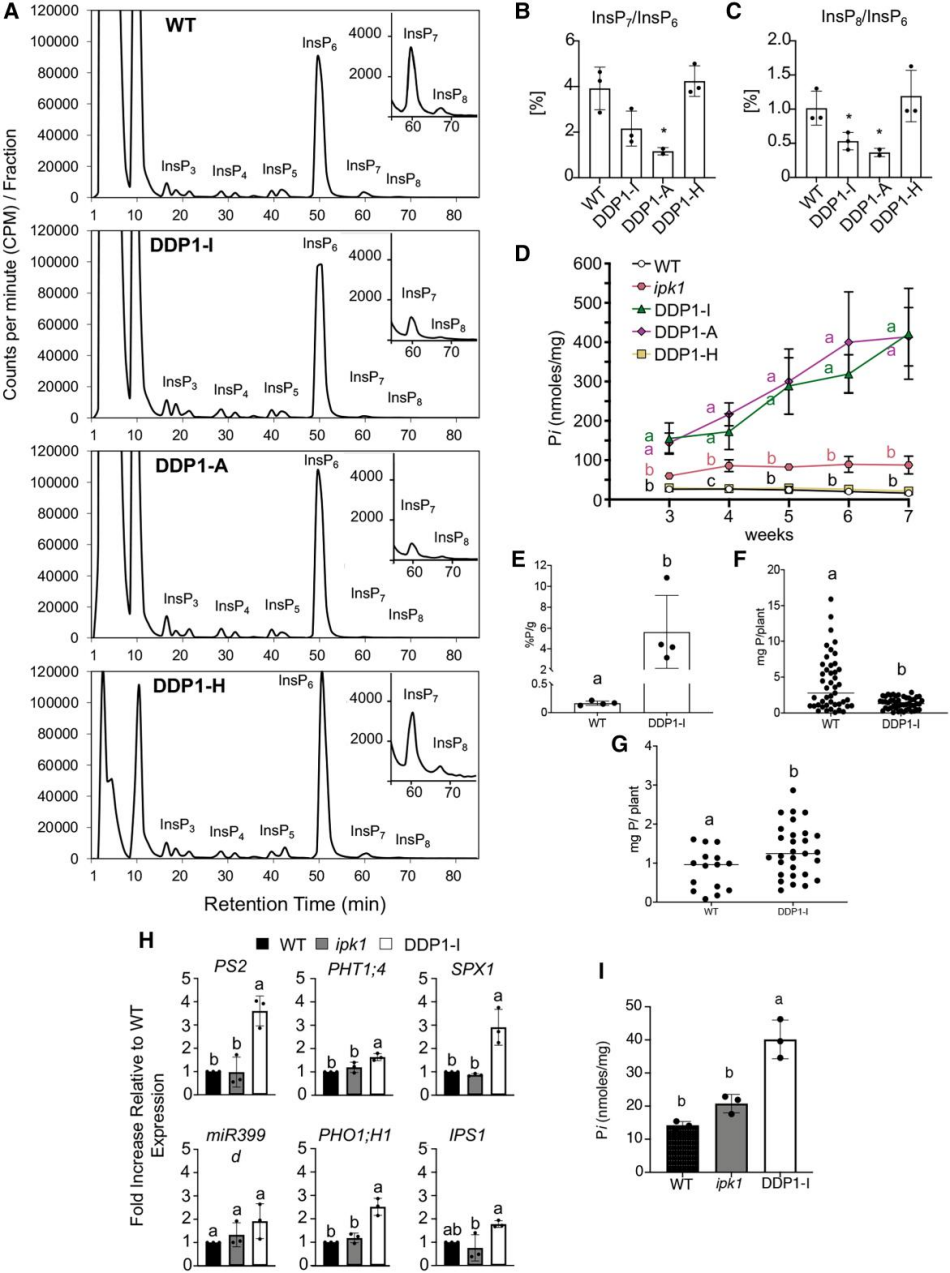
PP-InsP profiling, P*i* accumulation, and PSR gene expression in DDP1 OX transgenics. **A)** WT and DDP1 OX transgenics were grown for 14 d on semisolid 0.5× MS media with 0.2% agar then 100 *μ*Ci [^3^H]-*myo-*inositol was added for 4 d. All InsPs were extracted, separated using anion exchange HPLC, and data were analyzed as described in the “Materials and methods” section. These InsP profiles are representative of 2 to 3 independent replicates per genotype; see Supplementary Fig. S1 for all profiles. **B)** InsP_7_/InsP_6_ and **C)** InsP_8_/InsP_6_ ratios. Asterisks show significant differences from WT; analyzed using Student's *t*-test; *P < 0.05, error bars show Sd of *n* = 2 to 3. **D)** Leaf P*i* accumulation in soil-grown plants from 3 to 7 wk of growth. Each point represents pooled plant tissue from *n* = 2 to 4 independent experiments; error bars show Sd. Different letters indicate statistically significant means (Tukey honestly significant difference (HSD) test, *α* = 0.05). **E** to **G)** WT and DDP1 OX transgenics were grown for 100 d in hydroponic tracks in a greenhouse. **E)** Amount of P accumulated in shoot dry mass of WT and DDP1 OX transgenics. Each point represents the average of one track of *n* = 9 to 14 plants; error bars show Sd (Tukey HSD, *α* = 0.05). **F)** The amount of total P accumulated per individual plant regardless of total mass (WT *n* = 45; DDP1 Ox *n* = 49) and **G)** plants weighing 200 mg or less (WT *n* = 15; DDP1 Ox *n* = 29); (Tukey HSD, *α* = 0.05). **H)** PSR gene expression (relative to WT) and **I)** P*i* accumulation in whole seedlings grown for 10 d grown on media plates. Error bars denote Sd of *n* = 3 independent experiments. Different letters indicate statistically significant means (Tukey HSD, *α* = 0.05).

To determine whether DDP1 overexpression alters PP-InsP levels in planta, we measured InsPs and PP-InsPs in all 3 DDP1 OX lines and WT plants using *myo*-[^3^H] inositol radiolabeling (Desai et al. 2014; Adepoju et al. 2019). We observed a large decrease in InsP_7_ and InsP_8_ in DDP1-I and DDP1-A, whereas DDP1-H levels were similar to WT (Fig. 3A; Supplementary Fig. S1A). DDP1-A InsP_7_/InsP_6_ and InsP_8_/InsP_6_ ratios were significantly lower than WT ratios (Fig. 3, B and C). DDP1-I also had a significantly lower InsP_8_/InsP_6_ ratio compared to WT, although the InsP_7_/InsP_6_ ratio was not statistically significant from WT (Fig. 3B). Interestingly, there was no significant change in the InsP_7_/InsP_8_ ratio in the severe DDP1 OX lines compared to WT, suggesting that the decrease in InsP_7_ and InsP_8_ is proportional. Comparing the average percent of InsP_3_-InsP_8_ species in DDP1 OX transgenic lines to WT (Table 1) showed fluctuations in some InsP species; however, there were no notable changes in any species besides InsP_7_ and InsP_8_ across all replicates. Of all the InsP species, only InsP_7_ and InsP_8_ had a consistent decrease in DDP1 OX compared to WT. DDP1-I and DDP1-A, respectively, had 53 ± 9% and 25 ± 10% InsP_7_ of the total WT InsP_7_ pool and 51 ± 4% and 26 ± 11% of the total WT InsP_8_ pool. Taken together, the data indicate that DDP1 OX lines with higher DDP1-GFP protein accumulation have decreased InsP_7_ and InsP_8_, which correlates with the observed growth phenotypes of reduced plant size and leaf tip necrosis and chlorosis.

**Table 1.** Average percent of the total counts per minute for each InsP as a percentage compared to the total WT pool for the respective InsP species

	DDP1-H		DDP1-I		DDP1-A	
	Average %	Sd	Average %	Sd	Average %	Sd
InsP_3_	124%	± 42%	129%	± 31%	89%	± 6%
InsP_4_	118%	± 31%	144%	± 29%	157%	± 53%
InsP_5_	99%	± 21%	97%	± 1%	102%	± 46%
InsP_6_	94%	± 19%	102%	± 30%	108%	± 70%
InsP_7_	102%	± 17%	**53%**	**± 9%**	**25%**	**± 10%**
InsP_8_	122%	± 67%	**51%**	**± 4%**	**26%**	**± 11%**

Averages are of all replicates shown in Fig. 3A and Supplementary Fig. S1A. Values that represent ∼50% or less of the total WT pool are bolded.

### DDP1 overexpression impacts P*i* accumulation and PSR gene expression

Previous studies show that perturbing PP-InsPs can increase P*i* accumulation, which is associated with an upregulation of PSR genes (Kuo et al. 2014, 2018; Laha et al. 2019; Zhu et al. 2019; Land et al. 2021). As DDP1 OX phenotypes are P*i* dependent and similar to plants exhibiting symptoms of P*i* toxicity, we queried P*i* accumulation and expression of a specific set of PSR genes. We first gauged shoot P*i* accumulation in soil-grown DDP1 OX transgenics by measuring total shoot P*i* accumulation from 3 to 7 wk of growth (Fig. 3D). Both of the severe DDP1 OX lines had an average fold increase of 5.5 to 6 times more P*i* compared to WT after 3 wk of growth. Remarkably, P*i* accumulation ascended to a 25- to 26-fold increase after 7 wk of growth. This increase in P*i* accumulation in DDP1 OX was also significantly higher than what we observed in *ipk1* mutants (Fig. 3D). This is notable as *ipk1* mutants accumulated significantly higher P*i* compared to WT both in our experiments and in previous studies (Kuo et al. 2014, 2018; Land et al. 2021). DDP1-H transgenics showed no difference in P*i* accumulation compared to WT plants (Fig. 3D). Most strikingly, a comparison of *ipk1* and severe DDP1 OX line patterns of P*i* accumulation over time indicates that whereas P*i* accumulation in *ipk1* plants reaches a maximum at 3 wk, DDP1 OX plants continue to accumulate significantly elevated levels of P*i* over time. Given the differences in WT and DDP1 OX size, we were curious to know how much total phosphorus (P) was being absorbed by the plants. We queried the total P accumulation capacity of 100-d-old WT and DDP1-I plants grown under hydroponic conditions and reported that DDP1-I shoot tissue accumulated a significantly higher enrichment of P per gram compared to WT shoots (Fig. 3E). We also quantified the total P absorption on an individual plant basis. Overall, WT plants accumulated significantly higher levels of P on an individual plant basis compared to DDP1-I OX plants (Fig. 3F). Notably, WT plants of similar size to DDP1-I OX plants at the time of harvest (200 mg or less) showed significantly lower levels of P accumulation compared to DDP1-I OX transgenics. Taken together, these data suggest that while the current growth tradeoff of DDP1-I OX is not ideal for P absorption compared to WT plants with a larger mass, overexpression of DDP1 in *Arabidopsis* leads to a higher enrichment of P in the plant.

Given the increase in P*i* accumulation, we hypothesized that these increases were linked to increased expression of PSR genes. We queried a subset of PSR marker genes that play key roles in plant P*i* accumulation under P*i*-deplete conditions (*SPX1*, *PS2*, *miR399d*, *PHT1;4*, *PHO1;H1*, and *IPS1*) (Rouached et al. 2010; Jost et al. 2015; Chien et al. 2018). Of the genes queried, we observed that 10-d-old DDP1 OX whole seedlings showed a significant upregulation of *PS2*, *PHT1;4*, *SPX1*, and *PHO1;H1* compared to WT and *ipk1* seedlings (Fig. 3H). DDP1-I also had significantly increased *IPS1* compared to *ipk1*; however, this change was not significantly different from WT. *ipk1* seedlings trended toward increased expression of a few genes within the queried subset; however, these changes were not statistically significant. This result was expected as *ipk1* whole seedlings have not been shown to induce the PSR under replete conditions (Zhu et al. 2019), although it is important to consider that previous studies querying PSR gene expression specifically in *ipk1* roots show significant increases in PSR gene expression (Kuo et al. 2014, 2018). P*i* accumulation in these 10-d-old seedlings was also queried, and we found, as expected, that DDP1-I plants accumulated significantly more P*i* compared to WT and *ipk1* (Fig. 3I).

### NUDIX13 overexpression decreases PP-InsPs and increases P*i* accumulation

**Figure 4.**
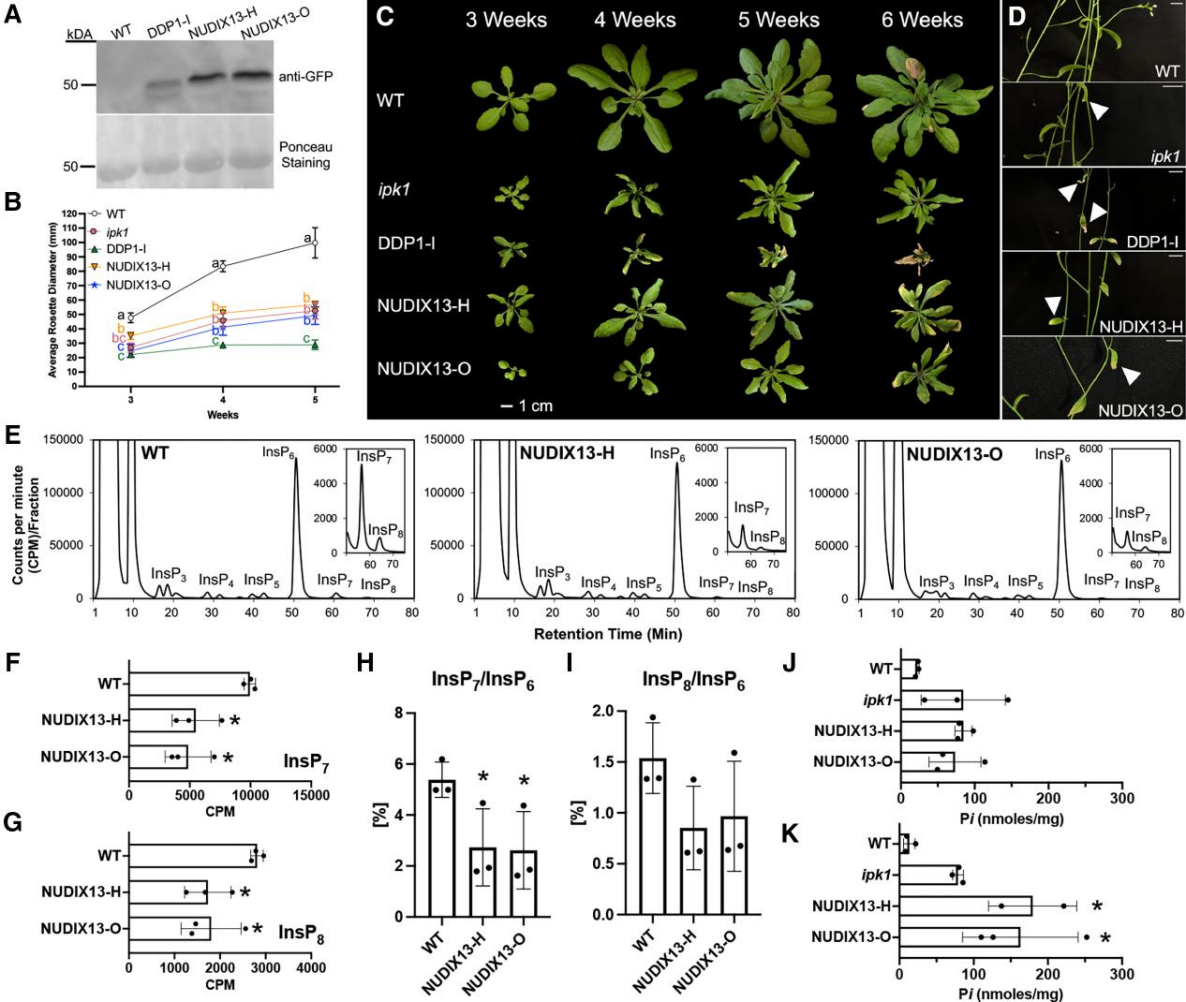
Characterization of NUDIX13 OX transgenics. **A)** Immunoblot of 3-wk-old leaf tissue from WT and selected NUDIX13 OX transgenics. Ponceau staining shows Rubisco accumulation in all plants as a positive control (∼50 to 56 kDa). **B)** Average rosette diameter over time. Each point represents *n* = 3 independent experiments with over 12 plants per genotype per experiment; error bars show Sd. Different letters indicate statistically significant means (Tukey honestly significant difference (HSD) test, *α* = 0.05). **C)***Arabidopsis* rosette growth over the course of 5 wk, each image representative of *n* = 4 to 5 independent experiments containing 12 or more plants per genotype. Images were digitally extracted for comparison. Scale bar = 1 cm. **D)** Close-up view of yellowing cauline leaves. White arrowheads indicate yellowing cauline leaves. Scale bar = 1 cm. **E)** HPLC analysis of WT and NUDIX13 OX transgenics, *n* = 3. See Supplementary Fig. S2 for additional profiles. **F)** Total InsP_7_, **G)** total InsP_8_, **H)** InsP_7_/InsP_6_, and **I)** InsP_8_/InsP_6_ ratios. Asterisks show significant differences from WT; analyzed using Student's *t*-test; *P < 0.05, error bars show Sd of *n* = 3. **J**, **K)** Leaf P*i* accumulation in soil-grown plants from 3 **J)** and 6 wk **K)** of growth. Analyzed using Student's *t*-test; *n* = 2 to 3 independent experiments; *P < 0.05, error bars show Sd.

We sought to determine if similar PP-InsP reductions and P*i* accumulation patterns could be achieved by overexpressing the native *Arabidopsis* enzyme, NUDIX13, given this enzyme shares predicted (Kraszewska 2008) and known substrates (Olejnik et al. 2007) with DDP1. Using the same strategy that we used for our stable DDP1 OX transgenics, NUDIX13-GFP transgenic plants were generated in the *Arabidopsis* Col-0 background with the CaMV 35S promoter. Two lines (NUDIX13-H and NUDIX13-O) were found to have high accumulation of NUDIX13-GFP (Fig. 4A) and were prioritized for our study. Similar to, yet less severe than the DDP1 OX transgenics, NUDIX13 OX plants had significantly smaller rosettes and exhibited leaf tip necrosis and chlorosis phenotypes (Fig. 4, B to D). NUDIX13-H and NUDIX13-O showed no significant difference from *ipk1* plants in terms of rosette diameter size but appeared to have larger zones of leaf tip necrosis and chlorosis on rosette and cauline leaves compared to *ipk1* (Fig. 4, C and D).

Quantification of PP-InsPs in NUDIX13-O and NUDIX13-H plants revealed significant decreases in InsP_7_ and InsP_8_ pools compared to WT plants (Fig. 4, E to G; Supplementary Fig. S2). NUDIX13-O and NUDIX13-H had significantly lower InsP_7_/InsP_6_ ratios compared to WT (Fig. 4H). InsP_8_/InsP_6_ ratios trended to be lower than WT but were not significant (Fig. 4I), and NUDIX13-O had significantly higher InsP_8_/InsP_7_ ratios compared to WT whereas NUDIX13-H trended to be higher but was not significant (Supplementary Fig. S2B). Based on these ratios and quantification of InsP species as compared to WT pools, the decrease in InsP_7_ and InsP_8_ appeared to be proportional in NUDIX13 transgenics (Fig. 4, F and G; Table 2). Comparing the average percent of InsP_3_-InsP_8_ species in all lines compared to WT revealed that there were no significant changes in any InsP species except for InsP_7_ and InsP_8_ (Table 2). NUDIX13-O and NUDIX13-H, respectively, displayed 35 ± 4% and 36 ± 0% InsP_7_ of the total WT InsP_7_ pool, and 48 ± 4% and 39 ± 2% of the total WT InsP_8_ pool.

**Table 2.** The average percent of total, individual InsP species compared to the total WT pool expressed as a percentage Averages for NUDIX13 OX were taken from all replicates shown in Fig. 4 and Supplementary Fig. 2 and nudix13-1 from Supplementary Fig. S3; *n* = 3 biological replicates per genotype

	NUDIX13-O		NUDIX13-H		nudix13-1	
	Average %	Sd	Average %	Sd	Average %	Sd
InsP_3_	91%	± 2%	109%	± 8%	96%	± 4%
InsP_4_	96%	± 4%	105%	± 13%	127%	± 4%
InsP_5_	92%	± 0%	102%	± 9%	139%	± 1%
InsP_6_	105%	± 3%	100%	± 3%	89%	± 1%
InsP_7_	**35%**	**± 4%**	**36%**	**± 0%**	207%	± 5%
InsP_8_	**48%**	**± 4%**	**39%**	**± 2%**	128%	± 15%

Values that represent ∼50% or less of the total WT pool are bolded. Values that represent ∼120% or higher of the total WT pool are underlined.

We also quantified PP-InsP accumulation in a loss-of-function *Arabidopsis* mutant carrying a T-DNA insertion in *NUDIX13*, designated here as *nudix13-1* (SALK_031617). In contrast to the reduction in InsP_7_ and InsP_8_ seen in NUDIX13 OX transgenics, *nudix13-1* mutants accumulated significantly higher levels of InsP_7_ and InsP_8_ as compared to WT plants (Supplementary Fig. S3; Table 2). *nudix13-1* mutants contained 207% ± 5% InsP_7_ and 128% ± 15% InsP_8_ of the WT PP-InsP pools (Table 2). Additionally, *nudix13-1* contained significantly higher levels of InsP_4_ and InsP_5_ and lower levels of InsP_6_ as compared to WT plants (Supplementary Fig. S3; Table 2). This demonstrates that disruption of *NUDIX13* alters plant InsP and PP-InsP profiles in a way consistent with a native function in hydrolyzing PP-InsPs.

Given decreased plant growth and decreased PP-InsP accumulation, we also measured P*i* accumulation in NUDIX13 OX shoot tissue. NUDIX13 OX lines had accumulated significantly higher levels of P*i* compared to WT after 3 and 6 wk of growth (Fig. 4, J and K). We report that there was no significant difference in P*i* accumulation in NUDIX13 OX lines compared to WT or *ipk1* mutants after 3 wk of growth (Fig. 4J). After 6 wk of growth, NUDIX13 OX lines accumulated significantly higher levels of P*i* compared to WT plants (Fig. 4K). Taken together, our data demonstrate that DDP1 OX and NUDIX13 OX lines are similarly impacted through decreased plant size, P*i* toxicity phenotypes, decreased PP-InsPs, and increased P*i* accumulation.

### NUDIX13 hydrolyzes PP-InsPs and other predicted substrates in vitro

**Figure 5.**
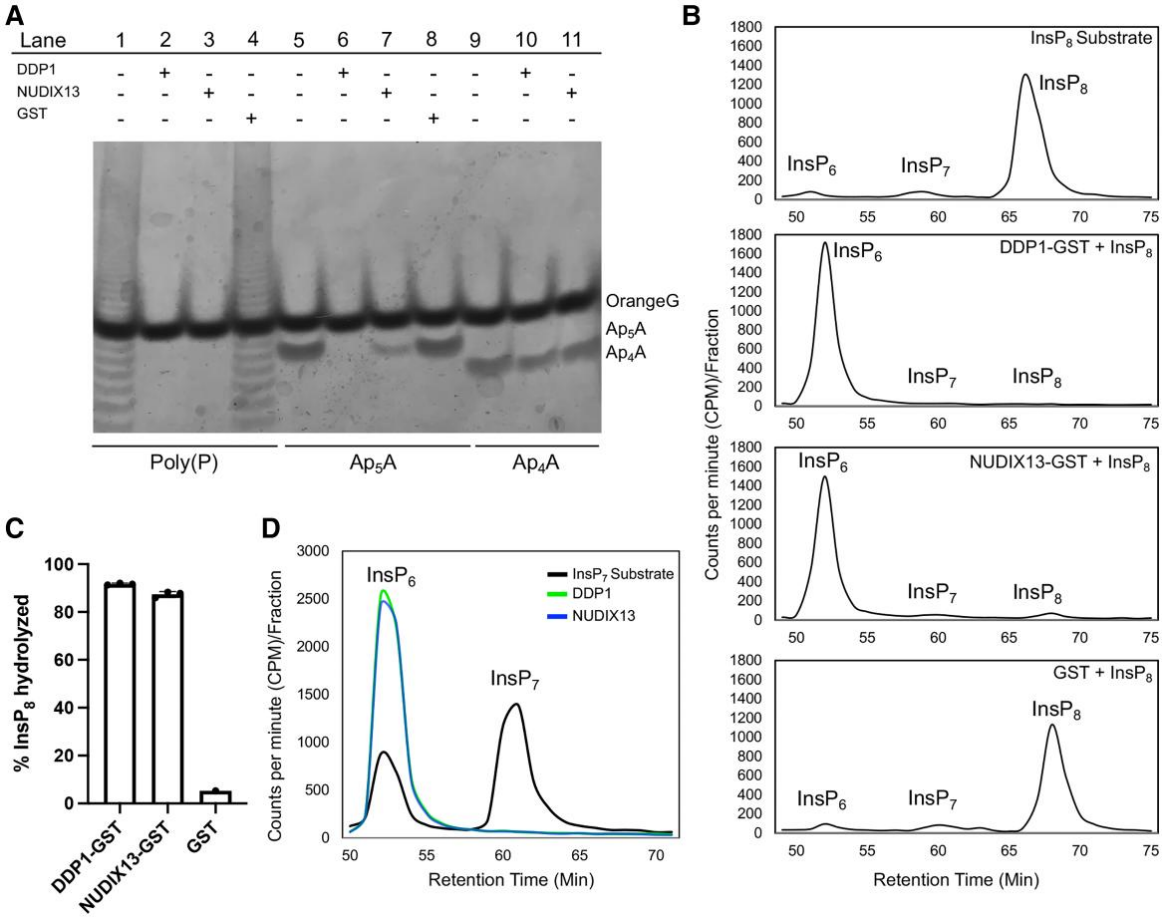
In vitro phosphatase assays with purified recombinant DDP1-GST and NUDIX13-GST. **A)** Purified DDP1-GST, NUDIX13-GST, or GST alone (negative control) was incubated with polyP (Lanes 1 to 4), Ap_5_A (Lanes 5 to 8), or Ap_4_A (Lanes 9 to 11) for 1.5 h at 37 °C, and then the reactions were resolved by PAGE and stained with Toluidine blue. Bands corresponding to Orange G (loading dye), Ap_5_A, and Ap_4_A are indicated on the right; undigested polyP appears ladderlike. Reactions in the absence (−) or presence (+) of enzyme or control GST protein are indicated in the table. **B** to **D)** DDP1-GST, NUDIX13-GST, or GST alone (negative control) was incubated with radiolabeled InsP_8_**B)** or InsP_7_**D)** for 1.5 h at 37 °C, and then the reaction products were resolved by HPLC and measured by liquid scintillation counting. Representative chromatograms with peaks corresponding to InsP_6_, InsP_7_, or InsP_8_ are shown. Reactions that lacked enzyme or GST control protein (traces labeled “InsP_7_/_8_ substrate”) show the starting substrate in each reaction. **C)** Percentage of InsP_8_ hydrolyzed by each enzyme (*n* = 3); error bars show Sd. **D)** HPLC analyses of enzymatically synthesized InsP_7_ substrate incubated with DDP1 or NUDIX13, or without enzyme, *n* = 1.

While our in planta data are indicative of NUDIX13 breaking down PP-InsPs, we sought to confirm this through in vitro phosphatase assays. We purified recombinant DDP1-GST, NUDIX13-GST, and GST (Olejnik et al. 2007; Ogawa et al. 2008; Márquez-Moñino et al. 2021) for our assays. GST was used as a negative control for substrate degradation. To ensure the purified enzymes were active, we used Ap_5_A as a positive control for hydrolysis and Ap_4_A as a negative control (Olejnik et al. 2007; Ogawa et al. 2008; Lonetti et al. 2011; Andreeva et al. 2019). We also included polyP as a substrate since DDP1 has been empirically shown to break down polyP in vitro (Lonetti et al. 2011; Andreeva et al. 2019). Our data indicate that purified DDP1-GST and NUDIX13-GST are active as shown through hydrolysis of Ap_5_A and polyP (Fig. 5A). Neither enzyme hydrolyzed Ap_4_A, which is consistent with previous reports. NUDIX13 is also capable of hydrolyzing polyP, indicating its similarity in substrate preferences as DDP1. To compare to our in planta PP-InsP data, InsP_7_ and InsP_8_ molecules were enzymatically synthesized, as previously described in Adepoju et al. (2019). DDP1-GST, NUDIX13-GST, and GST were then incubated with InsP_7_ and InsP_8_ for 1.5 h, separated using HPLC, and detected by scintillation counting (Desai et al. 2014; Adepoju et al. 2019). Our data demonstrate that DDP1 and NUDIX13 hydrolyze InsP_7_ and InsP_8_ in vitro, with roughly 90% InsP_8_ hydrolyzed after a 1.5-h incubation (Fig. 5, B and C). DDP1 and NUDIX13 also hydrolyzed InsP_7_ when incubated with an enzymatically synthesized InsP_7_ substrate (Fig. 5D). Collectively, our data demonstrate that DDP1 and NUDIX13 hydrolyze PP-InsPs, Ap_5_A, and polyP in vitro.

### DDP1-GFP and NUDIX13-GFP colocalize with PP-InsP synthetic enzymes

**Figure 6.**
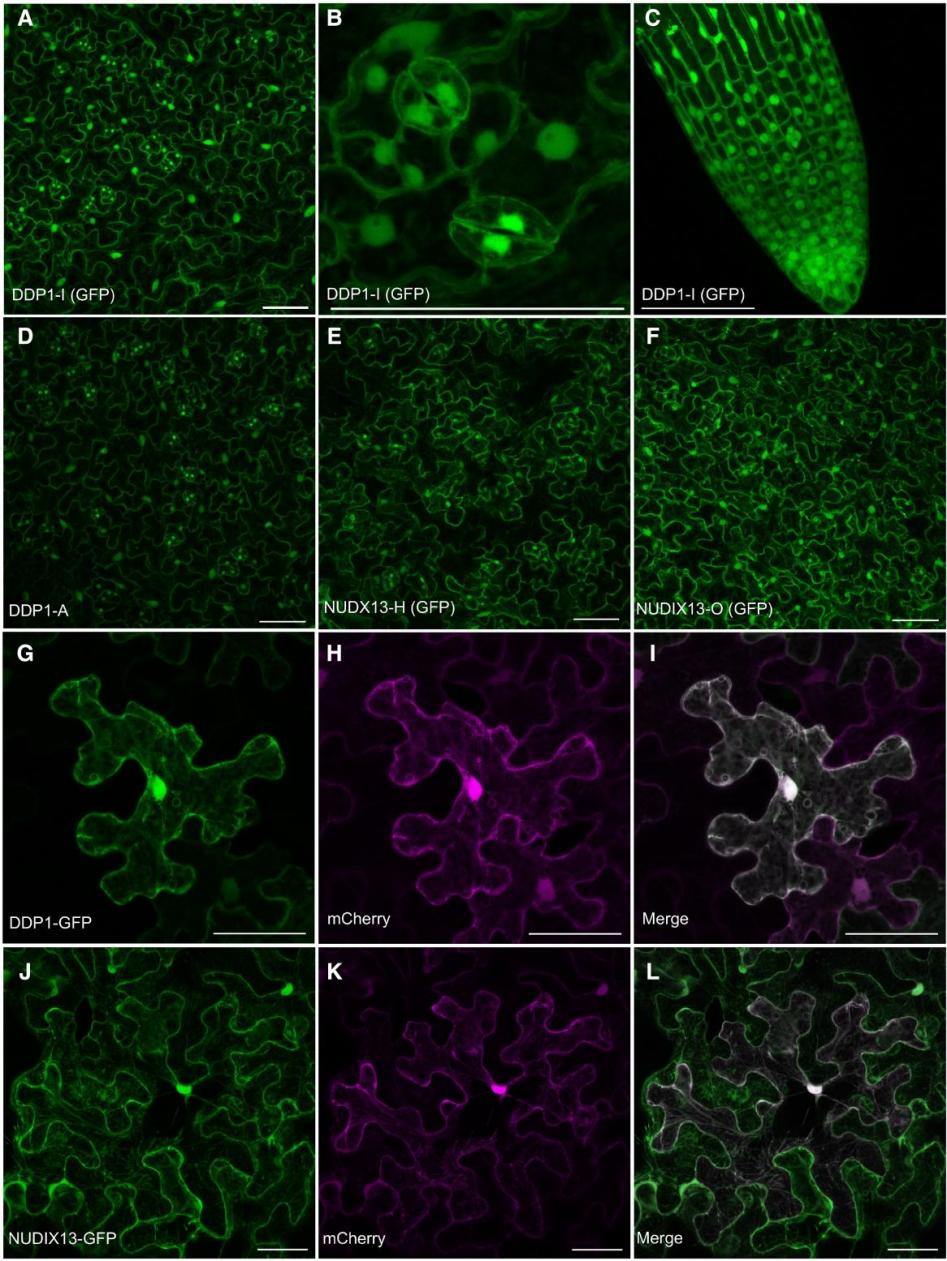
Confocal imaging of DDP1-GFP and NUDIX13-GFP expression in *Arabidopsis* and *N. benthamiana*. **A**, **B)***Arabidopsis* DDP1-I leaves and **C)** roots, **D)** DDP1-A leaves, **E)** NUDIX13-H leaves, and **F)** NUDIX13-O leaves. **G)** CaMV35S:DDP1-GFP and **J)** CaMV35S:NUDIX13-GFP transient expression in *N. benthamiana* leaves 48-h postinfiltration. *N. benthamiana* leaves coinfiltrated with DDP1-GFP **G** to **I)** or NUDIX13-GFP **J** to **L)** with unconjugated mCherry. DDP1-GFP and NUDIX13 are shown in green, and unconjugated mCherry is shown in magenta. All images are presented as maximum intensity projections from confocal *Z*-stack optical sections. All scale bars = 50 *µ*m.

Since DDP1 and NUDIX13 hydrolyze PP-InsPs in vitro and in planta, we sought to determine if the subcellular localization of DDP1 and NUDIX13 in planta was similar to the enzymes that synthesize PP-InsPs. While the native locations of PP-InsPs have yet to be determined in vivo, many of the PP-InsP synthesis enzymes in plants localize to the cytoplasm and nuclei (Xia et al. 2003; Kuo et al. 2018; Adepoju et al. 2019). Subcellular distribution of DDP1-GFP and NUDIX13-GFP under control of the CaMV 35S promoter was assessed using confocal microscopy in transgenic *Arabidopsis* lines and infiltrated *Nicotiana benthamiana* leaves. Both constructs localized predominantly to the cytoplasm and nuclei of *Arabidopsis* cells (Fig. 6, A to L). Consistent with our immunoblotting data, GFP signal was abundant in DDP1-I, DDP1-A, NUDIX13-H, and NUDIX13-O cells and undetectable in WT and DDP1-H (Fig. 6, A to F; Supplementary Fig. S4). GFP expression was also high in guard cell nuclei (Fig. 6B). We transiently coexpressed DDP1-GFP or NUDIX13-GFP in *N. benthamiana* with 2 plant organelle marker constructs: unconjugated mCherry, which localizes to the cytoplasm and nucleus (Adepoju et al. 2019), and endoplasmic reticulum (ER)-mCherry, a construct that localizes to the ER (Nelson et al. 2007). We also included YFP-DDP1 (DDP1 with an N-terminal YFP tag) to increase confidence that the localization pattern was not influenced by the position of the tag. DDP1-GFP, NUDIX13-GFP, and YFP-DDP1 colocalize substantially with unconjugated mCherry in the nucleus and the cytoplasm (Fig. 6, G to L). In contrast, we observed little if any colocalization with the ER-mCherry marker (Supplementary Figs. S5 to S7). YFP-DDP1 localized to the same compartments as DDP1-GFP, confirming that the pattern was not impacted by the position of the tag (Supplementary Fig. S7). Taken together, our data show that DDP1 and NUDIX13 both localize to the cytoplasm and nucleus in compartments, which is consistent with that of other PP-InsP synthesis enzymes.

### DDP1 overexpression in pennycress recapitulates *Arabidopsis* phenotypes

**Figure 7.**
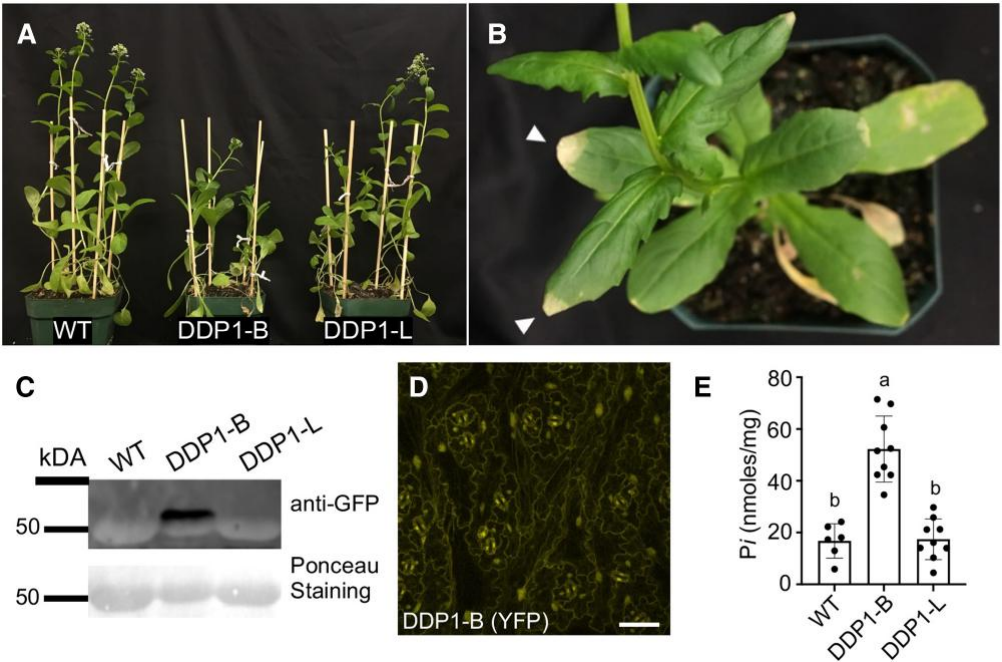
Characterization of pennycress DDP1 OX transgenics. **A)** Physiology of WT pennycress and 2 independent heterozygous DDP1 OX transgenics lines after 5 wk of growth. **B)** Close-up view of DDP1-B leaf tip necrosis and chlorosis after 7 wk of growth on soil. White arrowheads mark yellowing of leaf tips. **C)** Immunoblot of 6-wk-old leaf tissue from WT and heterozygous pennycress DDP1 OX lines. Ponceau staining shows Rubisco accumulation in all plants as a positive control (∼50 to 56 kDa). **D)** Stable YFP-DDP1 expression in pennycress DDP1-B leaves. Scale bar = 50 *µ*m. **E)** Shoot P*i* accumulation in 6- to 7-wk-old pennycress leaf tissue. Error bars denote Sd of *n* = 3 independent experiments; each point represents leaf tissue of an individual heterozygous plant. Different letters indicate statistically significant means (Tukey honestly significant difference (HSD) test, *α* = 0.05).

Based on the unique ability of DDP1 OX and NUDIX13 OX plants to accumulate higher amounts of P*i* over time, we sought to translate this into a strategy to develop a cover crop to recover excess P*i* from soil. For this study, we focused on DDP1 given its increased capacity for P*i* accumulation compared to NUDIX13 OX transgenics. We selected *T. arvense* (pennycress), a winter annual cover crop that is commonly used by farmers and shows promise as a biofuel crop (Chopra et al. 2020). Notably, recent strides have been made to establish pennycress as a model cover crop system through its recently sequenced genome and assembled transcriptome, and like *Arabidopsis*, it can be easily transformed by floral dipping (McCormick 2018), making it an ideal system for translation of our findings in *Arabidopsis*. We demonstrated that pennycress plants can be stably transformed to overexpress DDP1 and show similar phenotypes as *Arabidopsis* DDP1 OX transgenics (Fig. 7). It is important to note that we characterized heterozygotes in this study as our homozygous pennycress lines were severely growth compromised and displayed extreme P*i* toxicity (Supplementary Fig. S8). After 5 wk of growth, the DDP1-B OX line with higher transgenic protein accumulation manifested leaf tip necrosis and chlorosis phenotypes similar to what we observed in *Arabidopsis* (Fig. 7, A to C). Our pennycress DDP1 OX transgenics overexpress DDP1 with an N-terminal YFP tag, which accumulates in the same subcellular compartments (nuclear and cytosolic) as in *Arabidopsis* DDP1 OX plants and *N. benthamiana* leaf tissue (Fig. 7D). Strikingly, pennycress DDP1-B OX plants exhibit significantly higher P*i* accumulation compared to WT and the WT-like DDP1-L OX line that expresses undetectable levels of YFP-DDP1 (Fig. 7E). Taken together, these data demonstrate that overexpression of DDP1 in pennycress recapitulates many of the same phenotypes that we observed in *Arabidopsis*, including DDP1 subcellular localization, P*i* toxicity phenotypes, and increased P*i* accumulation in leaf tissue.

## Discussion

The identification of PP-InsP signaling components in plants, along with compelling evidence connecting PP-InsPs to P*i* sensing (Laha et al. 2015; Kuo et al. 2018; Adepoju et al. 2019; Laha et al. 2019; Zhu et al. 2019; Land et al. 2021; Laha et al. 2022) has yielded valuable genetic targets for P*i* phytoremediation approaches. Manipulation of the PP-InsP signaling pathway in plants offers an opportunity to address the P*i* crisis. In this work, we sought to use a synthetic biology approach to manipulate PP-InsPs in transgenic plants to facilitate the study of the long-term consequences of decreasing PP-InsPs on P*i* accumulation and to evaluate the preliminary translational potential of depleting PP-InsPs to increase P*i* accumulation in planta. While previous work explores the impact of VIP (Mulugu et al. 2007; Shears et al. 2017) and SIW14/DSP-PFA (Aceti et al. 2008; Wang et al. 2018; Gaugler et al. 2022) plant enzymes, we explored the role of 2 NUDIX enzymes and their impact on plant PP-InsP hydrolysis.

### Expression of a synthetic gene, DDP1, and a native gene, NUDIX13, allows plants to accumulate large amounts of P*i*

DDP1 OX transgenics with higher DDP1 protein accumulation created growth-compromised plants with P*i* toxicity phenotypes and negatively impacted reproductive phenotypes (Figs. 2 and 7). These distinct DDP1 OX phenotypes are P*i* dependent as growing DDP1 OX transgenics on depleted P*i* conditions rescued the leaf and seed phenotypes (Fig. 2, E to G). Notably, DDP1 OX overexpression decreases PP-InsPs (Fig. 3, A to C), increases PSR gene expression (Fig. 3H), and confers a unique P*i*-accumulating phenotype (Figs. 3, D to E, and 7E) with promising translational applications. To explore native plant PP-InsP phosphatases, we identified *Arabidopsis* NUDIX13 as an ideal enzyme given its similarities to DDP1 in structural homology (Olejnik et al. 2007) and predicted function (Kraszewska 2008). Our in vitro experiments demonstrate that recombinant DDP1 and NUDIX13 hydrolyze PP-InsPs, polyP, and Ap_5_A (Fig. 5). These results align with previous studies that assessed substrate hydrolysis of DDP1 (Safrany et al. 1999; Lonetti et al. 2011; Andreeva et al. 2019; Márquez-Moñino et al. 2021) and NUDIX13 (Olejnik et al. 2007; Ogawa et al. 2008).

Our data show that stable NUDIX13 expression in *Arabidopsis* is similar to DDP1 OX transgenics, as NUDIX13 OX transgenics were also smaller in size and displayed P*i* toxicity phenotypes (Fig. 4). Transgenics expressing high levels of DDP1 and NUDIX13 exhibited decreased PP-InsPs with a similar trend in proportionally reduced InsP_7_ and InsP_8_ levels (Figs. 3, A and B, and 4, E to I); however, NUDIX13 OX plants were less growth compromised compared to DDP1 OX plants (Fig. 4, B and C). Moreover, while NUDIX13 OX shoot tissue accumulated lower levels of P*i* compared to DDP1 OX plants, NUDIX13 OX plants had significantly higher levels of P*i* accumulation than WT after 6 wk of growth (Fig. 4K). Taken together, these data suggest that both DDP1 and NUDIX13 are similar enzymes that, when overexpressed in planta, can reduce PP-InsPs and increase plant P*i* accumulation.

### Overexpression of PP-InsP phosphatases results in unique biological characteristics distinct from PP-InsP kinase mutants

DDP1 and NUDIX13 overexpression in planta allowed us to achieve our original goal of reducing PP-InsPs. This strategy is unique from studying InsP and PP-InsP synthesis mutants. There are 3 types of InsP or PP-InsP synthesis mutants with defects in P*i* sensing that have been described in the current literature; the functions of the WT-encoded enzyme products of these mutants are shown in Fig. 1. *ipk1* mutants have reduced levels of PP-InsPs as IPK1 enzyme synthesizes PP-InsP precursor, InsP_6_ (Laha et al. 2015; Kuo et al. 2018; Land et al. 2021). Similarly, *itpk1* mutants also showed a reduction in InsP_7_ (Kuo et al. 2018) and InsP_8_ (Laha et al. 2022) compared to WT seedlings. *vih1-2vih2-4* double mutants were reported to have undetectable levels of InsP_8_ and double the WT pool of InsP_7_, in addition to a loss in viability (Zhu et al. 2019). All characterized mutants showed P*i*-dependent growth phenotypes, increases in shoot P*i* accumulation compared to WT plants, and an induction of PSR genes (Kuo et al. 2014, 2018; Zhu et al. 2019). This induction of the PSR is thought to result from the lack of InsP_8_ available within these mutants, which acts to release the PHR1 transcription factor.

While there are similarities in the way that PP-InsPs levels are altered in previously characterized PP-InsP synthetic mutants and the plants described in our work, there are key differences worth noting. First, DDP1 and NUDIX13 OX transgenics actively hydrolyze PP-InsPs in contrast to loss-of-function mutants that lack enzymes that synthesize InsP_6_ and/or PP-InsPs. We hypothesize that DDP1 and NUDIX13 in our transgenics are able to hydrolyze InsP_7_ and InsP_8_ faster than the PP-InsP synthesis enzymes are able to replenish PP-InsP pools (Fig. 1). This hypothesis draws from suggestions over the years that PP-InsP turnover is a cyclical interconversion between InsP_6_, InsP_7_ isomers, and InsP_8_ based on current evidence in yeast and humans (Kilari et al. 2013; Shears 2018; Dollins et al. 2020). Thus, we have likely tipped the delicate scale of this continual cycle of PP-InsP degradation and synthesis by increasing the phosphatases in the mix. Another key difference between our DDP1 OX plants and *ipk1* mutants is the level and duration of P*i* accumulation (Fig. 3D). While our work and that of others show that *ipk1* mutants tend to accumulate higher P*i* compared to WT plants (Stevenson-Paulik et al. 2005; Kuo et al. 2014, 2018; Land et al. 2021), DDP1 expression in plants allows for a much greater and continual accumulation of P*i* over time. This increased P*i* accumulation is associated with a greater induction of PSR genes in DDP1 as compared to *ipk1* seedlings (Fig. 3H). Notably, previous work on *ipk1* mutants measured PSR gene expression in roots (Kuo et al. 2014, 2018), while our work has focused on the whole plant impact of altering PP-InsPs, as above ground tissues present the greatest opportunity with regard to future phytoremediation strategies.

### DDP1 and NUDIX13 functionality in planta

Overexpression of NUDIX13 in *Arabidopsis* yields similar results as overexpressing DDP1 in plants, including a reduction in PP-InsPs (Figs. 3, A to C, and 4, E to G) and an increase in P*i* accumulation (Figs. 3D, 4K, and 7E). Two likely important factors for NUDIX13 to lead to these changes when overexpressed are its substrate specificity and its ability to colocalize with potential substrates. Regarding substrate specificity, our in vitro data show the DDP1 and NUDIX13 can hydrolyze the same substrates (PP-InsPs, polyP, and Ap_5_A) (Fig. 5). Our study demonstrates *Arabidopsis* NUDIX13 can hydrolyze PP-InsPs in vitro. A previous study from Olejnik et al. (2007) concluded that NUDIX13 was unable to hydrolyze InsP_7_ under their reaction conditions. It is possible that this discrepancy between our results is due to our use of a more sensitive technique to quantify PP-InsP hydrolysis. It is also important to note that NUDIX13 activity on polyP had not been empirically shown in the literature before this study while other studies demonstrate DDP1 hydrolyzes polyP (Lonetti et al. 2011; Andreeva et al. 2019). We showed that overexpression of both NUDIX13 and DDP1 results in decreased PP-InsPs with a similar trend in proportionally reduced InsP_7_ and InsP_8_ levels (Figs. 3, A to C, and 4, E to I). Given what is known about this class of enzymes, it seems likely that both enzymes act on 1-InsP_7_, 5-InsP_7_, and InsP_8_. However, because our measurements cannot distinguish between different isomers of PP-InsPs, we cannot say with certainty that both 1- and 5-PP-InsPs are acted on by these enzymes. It is important to note that NUDIX13 OX plants display less compromised growth as compared to DDP1 OX plants (Figs. 2, A to C, and 4, B and C), yet NUDIX13 OX leaves accumulate more P*i* at a later stage of growth compared to WT plants (Fig. 4, J and K). Conversely, *nudix13-1* mutants showed increased levels of PP-InsPs and alterations in InsPs compared to WT (Supplementary Fig. S3; Table 2). These data support the role of NUDIX13 as a native plant PP-InsP phosphatase. Since there are 27 known and annotated NUDIX enzymes in *Arabidopsis* (Ogawa et al. 2008), there are likely additional NUDIX enzymes with the potential to impact P*i* sensing in plants. Future studies exploring the other NUDIX enzymes will be important to expand our understanding of plant PP-InsP regulation.

Another key finding of DDP1 and NUDIX13 activity in plants stems from our localization data showing both enzymes localize to the nucleus and cytoplasm (Figs. 6 and 7D). While the specific locations of PP-InsPs have yet to be directly determined, DDP1-GFP and NUDIX13-GFP constructs show the same localization patterns as other known InsP and PP-InsP synthetic enzymes (Xia et al. 2003; Kuo et al. 2018; Adepoju et al. 2019), which is also consistent with the native DDP1 localization in yeast (Huh et al. 2003). Interestingly, NUDIX13 was originally annotated as a mitochondrial enzyme and 1 study shows NUDIX13 is located within the mitochondria in yeast and in *Arabidopsis* suspension cells (Olejnik et al. 2007), suggesting a potentially complicated compartmentalization of NUDIX substrates across eukaryotes.

Given the ability of both DDP1 and NUDIX13 to act on other substrates, it is reasonable to ask whether the hydrolysis of these molecules is important in our plants. These alternative substrates include 2 distinct types of molecules: polyP and Ap_n_As (specifically, Ap_5_A and Ap_6_A), which are known substrates for DDP1 in *S. cerevisiae* (Safrany et al. 1999; Nelson et al. 2007; Lonetti et al. 2011; Márquez-Moñino et al. 2021). Both molecular classes have also been linked to maintaining P*i* metabolism and cellular homeostasis in a variety of prokaryotic and eukaryotic organisms (Lorenzo-Orts et al. 2020; Pietrowska-Borek et al. 2020). Notably, polyP plays an important role in P*i* sensing and storage for yeast and protozoans (Secco et al. 2012; Vila et al. 2022) and yeast polyP synthesis is controlled by PP-InsPs (Guan et al. 2023). However, there is no evidence to suggest that these other potential substrates are present in plants. Studies report careful efforts made to detect and quantify these molecules in land plants and were unsuccessful (Pietrowska-Borek et al. 2011; Zhu et al. 2019; Lorenzo-Orts et al. 2020). Given this, we hypothesize that the most likely action of DDP1 and NUDIX13 expressed in plants is to deplete the PP-InsP pool, resulting in the observed impacts on P*i* accumulation and toxicity. If true, this would point to key differences between plants, yeast, and animals regarding these signaling molecules and the roles they play in vivo.

### The demand for translation: from studying PP-InsPs to implementing agricultural practices

The P*i* crisis is a complicated issue that will require a variety of innovative strategies to circumvent both P*i* deficiency (potential shortages and P*i*-deficient soils) and P*i* surplus (watershed pollution and lands saturated with P*i*). As the global population increases, there is a need for farmers to increase fertilizer inputs as crop production also increases. Between global food demand and higher fertilizer usage in urban areas, there is a subsequent increase in fertilizer runoff into the watersheds. One strategy that can be used is P*i* phytoremediation by leveraging plants to absorb excess P*i* from polluted water and soil. DDP1 overexpression in both a model species and cover crop provides unique germplasm to study how decreased PP-InsPs impact plant growth, physiology, and P*i* accumulation. This study synthetically modulated PP-InsPs to increase P*i* accumulation. A past study similarly targeted P*i* phytoremediation through plant P*i* accumulation by targeting PHR1, the transcription factor targeted by SPX1 and InsP_8_, in 3 ornamental garden plant species to reclaim P*i* from hydroponic solutions (Matsui et al. 2013). Matsui et al. demonstrated that overexpression of PHR1 in torenia, an ornamental plant, exhibited a leaf phenotype that was similar to DDP1 OX leaves. Another previous strategy to reduce P*i* watershed pollution has targeted key genes in the InsP_6_ synthesis pathway to limit InsP_6_ content in seeds and grains. This strategy is important as nonruminant livestock animals cannot digest InsP_6_ and so most is excreted as waste, which can pollute watersheds due to agricultural runoff (Sharpley and Withers 1994; Abelson 1999; Raboy 2002).

As we continue to learn more about the roles of PP-InsPs in plant P*i* sensing and growth, this will generate knowledge for developing unique strategies, ranging from P*i* removal from polluted environments to possibly improving P*i*-use efficiency. Pennycress DDP1 OX transgenics are an exciting proof of concept for a P*i* phytoremediation plant given the enhanced ability of DDP1-B OX transgenics to absorb P*i* from soil into leaf tissue (Fig. 7). DDP1 expression in the pennycress background was effective in P*i* accumulation in that working with the homozygous DDP1-B OX generation proved to be difficult based on the strong P*i* toxicity symptoms (Supplementary Fig. S8). The growth tradeoff we observed when expressing DDP1 is not desirable. This was evident in our data showing that individual WT plants had the capacity to absorb significantly higher levels of P compared to DDP1 OX transgenics (Fig. 3F). However, we also note that WT plants similar in size to DDP1 OX plants accumulated significantly lower levels of P compared to DDP1 OX plants (Fig. 3G). While the current negative growth tradeoff we observe in DDP1 OX plants is undesirable, it should not be overlooked that DDP1, if expressed in a regulated fashion, could be useful for future P*i* reclamation. The strategy we report here represents a step in altering PP-InsPs in planta for continual accumulation of P*i* from soil, a key step for future development of unique plants to remediate P*i*-polluted environments.

## Materials and methods

### Cloning and transformation

*DDP1* (YOR163w) from *S. cerevisiae* and *NUDIX13* (At3g26690) from *Arabidopsis thaliana* were PCR amplified for ligation into Gateway pENTR/D-TOPO entry vector. Using the Gateway LR Clonase II kit (Invitrogen Corp., Carlsbad, CA, USA), pENTR clones were recombined with Gateway plant destination vectors pK7FWG2 (C-terminus DDP1-GFP and NUDIX13-GFP) (VIB UGent Center for Plant Systems Biology). *Escherichia coli* transformed with these constructs was selected on antibiotic media. Plasmids purified from resulting colonies were sequencing for verification of correct cloning. *Agrobacterium tumefaciens* (strain GV3101) was transformed with these constructs and was used for transient *N. benthamiana* experiments or to transform *Arabidopsis* (ecotype Col-0) plants. All accession numbers and primer sets are available in Supplementary Table S1.

### Plant growth conditions

#### Growth chamber

All *Arabidopsis* plants were grown on soil under long-day conditions (16-h light/8-h dark, 55% humidity, day/night temperature 23/21 °C, 120 *µ*mol m^−2^ s^−1^). For radiolabeling experiments, seeds were sterilized using 100% ethanol for 1 min, transferred to a 30% (*v*/*v*) Clorox solution for 5 min and washed 5 times with ddH_2_O. Seeds were placed in 0.5× MS media + 0.2% agar and stratified for 3 d at 4 °C. Seedlings were transferred multiwell plates containing semisolid media (0.2% agar).

#### Greenhouse

*Arabidopsis* seeds were added to 3.2-cm square media cubes (Aeromax, Oasis Grower Solutions, Kent, OH, USA) saturated with a nutrient solution containing 230-mg/L Hydro-Gro Leafy (CropKing, Lodi, OH, USA). After 1 wk of growth, cubes were transferred into 122-cm nutrient film channels (CropKing) in a completely randomized design, comparing WT to DDP1 OX, 4 total replicates of 3 nutrient film tracks containing 18 plants fed by 1 water reservoir. Plants were initially watered with 479-mg/L Hydro-Gro Leafy at a flow rate of 0.05 mL/s, pH 6.0 ± 0.5. After 2 wk of growth, the restriction of nutrient flow was removed, and the pump was turned off for 16 h/d (between 16:00 and 8:00).

### Immunoblots of GFP-fusion proteins

Immunoblots were performed as previously reported (Burnette et al. 2003). Leaf tissue from soil-grown plants from was pulverized in liquid nitrogen and proteins were separated from cell debris. SDS-bromophenol blue loading dye was added to the extracted proteins and boiled for 5 min at 85 °C. After a subsequent centrifugation, the supernatant was loaded onto a polyacrylamide gel; equal amounts of protein were added from each sample. For immunoblotting, a 1: 5,000 dilution of anti-GFP antibody (Invitrogen Molecular Probes, Eugene, OR, USA) and a 1: 2,000 dilution of secondary goat anti-rabbit horseradish peroxidase antibody (Bio-Rad Laboratories, Hercules, CA, USA) were used to detect GFP.

### Subcellular localization and imaging

All *Arabidopsis* and *N. benthamiana* cells were imaged using a Zeiss LSM 880 confocal microscope (Carl Zeiss, Thornwood, NY, USA) equipped with a 25× C-Apochromat water immersion lens. *N. benthamiana* plant leaves were infiltrated with transformed with *A. tumefaciens*, as previously described (Kapila et al. 1997). Specifically, *Agrobacterium* cells were grown overnight, pelleted, and resuspended in MMA (10 mm MES, 10 mm MgCl_2_, 200 *µ*m acetosyringone) solution at an OD_600_ of 1.0. After a 2- to 4-h incubation period in the dark, *N. benthamiana* leaves were infiltrated with the *Agrobacterium* MMA cultures. Leaf sections were imaged after 24-, 48-, and 72-h postinfiltration. GFP was excited using a 488-nm argon laser and its fluorescence was detected using a 500- to 550-nm band-pass emission filter. GFP-tagged proteins were colocalized with a set of mCherry-tagged organelle markers (Nelson et al. 2007), mCherry was imaged using excitation with a HeNe 594-nm laser, and fluorescence was detected using a 600- to 650-nm band-pass emission filter. Chlorophyll autofluorescence was excited using a HeNe 594-nm laser, and emission above 650 nm was collected.

### InsP and PP-InsP accumulation of *Arabidopsis* seedlings

WT and NUDIX13 OX transgenics were grown in semisolid 0.5× MS media and 0.2% agarose for 2 wk, 30-*μ*E light intensity. Twenty-four seedlings of each genotype were transferred to Eppendorf tubes containing 300 *μ*L of 0.5× MS media, 0.2% agar, and 50 *μ*Ci of [^3^H] *myo*-inositol (20 Ci/mmol; American Radiolabeled Chemicals [ARC], St. Louis, MO, USA) for 4 d. InsPs were extracted from seedlings by pulverizing tissue in extraction buffer (1 m perchloric acid [HClO_4_], 3 mm EDTA, and 0.1 mg/mL InsP_6_) and vortexed with glass beads. The pH of the extract was neutralized to pH 6 to 8 using a neutralization buffer (3 mm EDTA and 1 m K_2_CO_3_). Samples were dried to a volume of 80 to 100 *μ*L. Negatively charged species were separated via HPLC using a 125 × 4.6 mm Partisphere SAX column (Sepax Technologies, Delaware, USA) and an ammonium phosphate elution gradient (Azevedo and Saiardi 2006). Radioactivity in all collected fractions was quantified using a scintillation counter.

### P*i* and P accumulation assays

P*i* was extracted from ∼50 mg from *Arabidopsis* and pennycress tissue of various ages. Samples were pulverized in liquid nitrogen. A 1:10 ratio of 1% acetic acid was added to each sample, which was vortexed and incubated on ice and centrifuged. Assays were performed on P*i* extracts using a modified microtiter assay as previously reported (Ames 1966). Fifty microliters of the supernatant and 1 mL of working reagent (5% *w*/*v* FeH_14_O_11_S·7H_2_O, 1% *w*/*v* (NH_4_)_2_MoO_4_, and 1N H_2_SO_4_ aq.) were incubated for an hour. Samples were placed in plastic cuvettes and absorbance at 660 nm was measured using a plate spectrophotometer. All P*i* concentrations were calculated based on a standard curve from a set of standards made with known P*i* concentrations. To determine total P levels, shoot mass of hydroponically-grown plants was harvested after 100 d and dried at 60 °C. Total P content was commercially analyzed at Brookside Laboratory (Bremen, OH, USA) using a nitric acid and hydrogen peroxide digestion using a MARS microwave (CEM Corporation, Mathews, NC, USA). P contents were analyzed on a 6500 Duo ICP (Thermo, Waltham, MA, USA).

### RNA isolation and quantitative real-time PCR

RNA was extracted from 10-d-old seedlings grown on 0.5× MS media plates using a Plant RNeasy kit with on column DNase treatment (Qiagen, Germantown, MD, USA). cDNA was generated using the iScript cDNA Synthesis Kit (Bio-Rad Laboratories, Hercules, CA, USA) and 1 *μ*g of total RNA. Quantitative PCR was performed using ∼20 ng of cDNA with PSR-specific primers and Applied Biosystems SYBR Green Universal Master Mix (Fisher Scientific, Waltham, MA, USA). All primer sequences used in the analysis can be found in Supplementary Table S1. All reactions were carried out in triplicate. Relative expression was calculated using the ΔΔCT method (Schmittgen and Livak 2008). Expression data were normalized to *PEX4* and relative to the fold change in WT.

### Expression and purification of DDP1- and NUDIX13-GST-tagged enzymes

#### DDP1

Gateway pENTR/D-TOPO entry vector containing DDP1 was recombined with Gateway destination vector pDEST15 (N-terminus GST; Invitrogen, Carlsbad, CA, USA) using the Gateway LR Clonase II kit (Invitrogen). After transformation of *E. coli* and selection on Carbenicillin LB plates, plasmids were purified, and sequences were verified. BL21 DE3 Turbo competent *E. coli* cells (Gelantis Biotechnology, San Diego, CA, USA) were transformed to with plasmid GST-DDP1. Single bacterial colonies expressing these constructs were picked from LB agar plates containing 50-*µ*g/mL carbenicillin and used to inoculate 5-mL liquid LB containing the same antibiotic concentration. This was shaken at 37 °C overnight. The following day, the culture was used as the inoculum for 250-mL LB/carb media (OD_600_ = 0.05). The culture was shaken at 37 °C until the OD_600_ = 0.4. Expression of DDP1 was induced with 0.1 mm IPTG (final concentration) for 4 h. Bacteria were pelleted at 5,000 × *g*, for 5 min at 4 °C. The pellet was washed with 1× protein buffered saline (PBS), pH 7.3, centrifuged again, and stored at −80 °C.

#### DDP1 protein purification

A frozen pellet from 125-mL induced culture was resuspended in 6-mL lysis buffer (20 mm Tris-HCl pH 7.5, 2 mm DTT, 150 mm NaCl, 0.5% Triton X-100, 150 *µ*m PMSF, 300-*µ*L bacterial protease inhibitor [Sigma P8465], 1-mg/mL lysozyme, and 3-*µ*L 5-*µ*g/mL DNase1) for 30 min before sonicating on ice 5 times for 30 s each with 60 s in between. The lysate was centrifuged at 22,500 × *g* for 20 min. The lysate supernatant was rocked overnight at 4 °C with 0.4-mL glutathione sepharose beads (Pharmacia) that had been equilibrated with 20 mm Tris-HCl pH 7.5, 2 mm DTT, and 150 mm NaCl. The beads were collected by gentle centrifugation at 400 × *g* for 5 min. The beads were washed 3 times with 20 mm Tris-HCl pH 7.5, 2 mm DTT, and 150 mm NaCl. DDP1 was eluted from the beads with 400-*µ*L successive elutions with 5 mm glutathione in the Tris/NaCl/DTT solution. Fraction #2 had the highest concentration of purest protein and was used for enzyme assays.

#### NUDIX13

Gateway pENTR/D-TOPO entry vector containing NUDIX13 was recombined with Gateway destination vector pDEST15 (Invitrogen, N-terminus GST) using the Gateway LR Clonase II kit (Invitrogen Corp., Carlsbad, CA, USA), and GST-NUDIX13 sequence was verified as above. Rosetta2 competent *E. coli* cells (Invitrogen) were transformed with plasmid GST-NUDIX13. Single bacterial colonies expressing these constructs were picked from LB agar plates containing 50-*µ*g/mL carbenicillin and 20-*µ*g/mL chloramphenicol. This was used to inoculate 5-mL liquid LB containing the same antibiotic concentrations and shaken at 37 °C overnight. The following day, the culture was used as the inoculum for 250-mL LB/carb/chlor media (OD_600_ = 0.05). The culture was shaken at 37 °C until the OD_600_ = 0.7. Expression of DDP1 was induced with 1 mm IPTG (final concentration) for 3 h. Bacteria were pelleted at 5,000 × *g*, for 5 min at 4 °C. The pellet was washed with 1× PBS, pH 7.3, centrifuged again, and stored at −80 °C.

#### NUDIX13 protein purification

A frozen pellet from 125-mL induced culture was resuspended in 6-mL lysis buffer (1× PBS, pH 7.3, 1 mm DTT, 0.5% Triton X-100, 150 *µ*m PMSF, 1-mg/mL lysozyme, and 3-*µ*L 5-*µ*g/mL DNase1) for 30 min before sonicating on ice 5 times for 30 s each with 60 s in between. The lysate was centrifuged at 22,500 × *g* for 20 min. The lysate supernatant was rocked overnight at 4 °C with 0.4-mL glutathione sepharose beads (Pharmacia) that had been equilibrated with 1× PBS, pH 7.3, 1 mm DTT, and 0.5% Triton X-100. The beads were collected by gentle centrifugation at 400 × *g* for 5 min. The beads were washed 3 times with 50 mm Tris-HCl pH 8.0, 350 mm NaCl, and 1 mm DTT. NUDIX13 was eluted from the beads with 400-*µ*L successive elutions with 5 mm glutathione in the Tris/NaCl/DTT solution. Fraction #2 had the highest concentration of purest protein and was used for enzyme assays.

### Ap_5_A and polyP hydrolysis enzyme assays

Ap_4_A (D4022), Ap_5_A (D1262), and polyP were purchased from Sigma. Fifty-microliter reactions containing 14-*μ*g purified NUDIX13-GST were incubated with 1 mm DTT, 100 mm Tris-HCl (pH 8.5), 5 mm MgCl_2_, and 0.68 mm Ap_4_A/Ap_5_A or 50-*μ*g polyP (Olejnik et al. 2007). These conditions were the same for DDP1-GST hydrolysis assays except 50 mm HEPES (pH 7.0) and 50 mm KCl were used instead of Tris-HCl (modified from Lonetti et al. 2011). All reactions were incubated at 37 °C for 30 min and chemically inactivated using 0.25 m EDTA and placed on ice. Once terminated, 5 *µ*L of 6XOrangeG dye was added to each sample. Samples were loaded onto phytate gels (35.5% acrylamide and 10× TBE) (Losito et al. 2009). Phytate gels were run overnight at 400 V, stained using toluidine blue staining solution (0.05% toluidine blue, 20% methanol, 2% glycerol, and 20 mm Tris-HCl pH 6.8 to 7.0) for 2 min, and destained with H_2_O for 30 s to 5 min.

### PP-InsP hydrolysis enzyme assays

Substrates for PP-InsP hydrolysis enzyme assays were synthesized using the in vitro substrate synthesis system from commercially available [^3^H] Ins(1,4,5)P_3_ (17.1 Ci/mmol; ARC, St. Louis, MO, USA) and purified enzymes as described in Adepoju et al. (2019). Enzyme activity assays were performed according to Olejnik et al. (2007) with the following modification: DDP1, NUDIX13, and GST assays were performed in a 50-*µ*L reaction at 37 °C for 30 min using 40 *μ*g of purified enzyme.

### Accession numbers

Sequence data and accession numbers from this article can be found in Supplementary Table S1.

## Supplementary Material

kiae582_Supplementary_Data

## Data Availability

The data underlying this article are available in the article and in its online supplementary material.

## Supplementary data

The following materials are available in the online version of this article.

**Supplementary Figure S1.** Additional InsP and PP-InsP profiles of DDP1 OX transgenics.

**Supplementary Figure S2.** Additional InsP and PP-InsP profiles of NUDIX13 OX transgenics.

**Supplementary Figure S3.** InsP and PP-InsP profiles of *nudix13-1* plants.

**Supplementary Figure S4.** DDP1-GFP and NUDIX13-GFP localization in *Arabidopsis* epidermal cells.

**Supplementary Figure S5.** Transient DDP1-GFP expression after 24 and 48 h.

**Supplementary Figure S6.** Transient NUDIX13-GFP expression in *N. benthamiana* leaves 24- and 48-h postinfiltration.

**Supplementary Figure S7.** *N. benthamiana* leaves co-infiltrated with YFP-DDP1, unconjugated mCherry, and ER-mCherry.

**Supplementary Figure S8.** Homozygous pennycress DDP1-B OX plants.

**Supplementary Table S1.** Oligonucleotides used in this study.

## Dive Curated Terms

The following phenotypic, genotypic, and functional terms are of significance to the work described in this paper:

PHR1 Gramene: AT4G28610
PHR1 Araport: AT4G28610

VIP2 Gramene: AT5G59710
VIP2 Araport: AT5G59710

PEX4 Gramene: AT5G25760
PEX4 Araport: AT5G25760

SPX1 Gramene: AT5G20150
SPX1 Araport: AT5G20150

PHR2 Gramene: Os07g25710
PHR2 Araport: Os07g25710

IPS1 Gramene: AT3G09922
IPS1 Araport: AT3G09922

miR399d Gramene: AT2G34202
miR399d Araport: AT2G34202

PHT1;4 Gramene: AT2G38940
PHT1;4 Araport: AT2G38940

DDP1 Gramene: YOR163W
DDP1 Araport: YOR163W

NUDIX13 Gramene: AT3G26690
NUDIX13 Araport: AT3G26690

VIP1 Gramene: AT1G43700
VIP1 Araport: AT1G43700

PS2 Gramene: AT1G73010
PS2 Araport: AT1G73010

PHO1;H1 Gramene: At1g68740
PHO1;H1 Araport: At1g68740

IPK1 Gramene: AT5G42810
IPK1 Araport: AT5G42810

ITPK1 Gramene: AT5G16760
ITPK1 Araport: AT5G16760
