# Supplementary material for: Using native and synthetic genes to disrupt inositol pyrophosphates and phosphate accumulation in plants
Source: Plant Physiol. 2024 Oct 30;197(1):kiae582. doi: 10.1093/plphys/kiae582 (PMC11663554; doi:10.1093/plphys/kiae582)
Supplement: kiae582_Supplementary_Data [file kiae582_supplementary_data.zip › SupplementaryData.pdf]

(a)

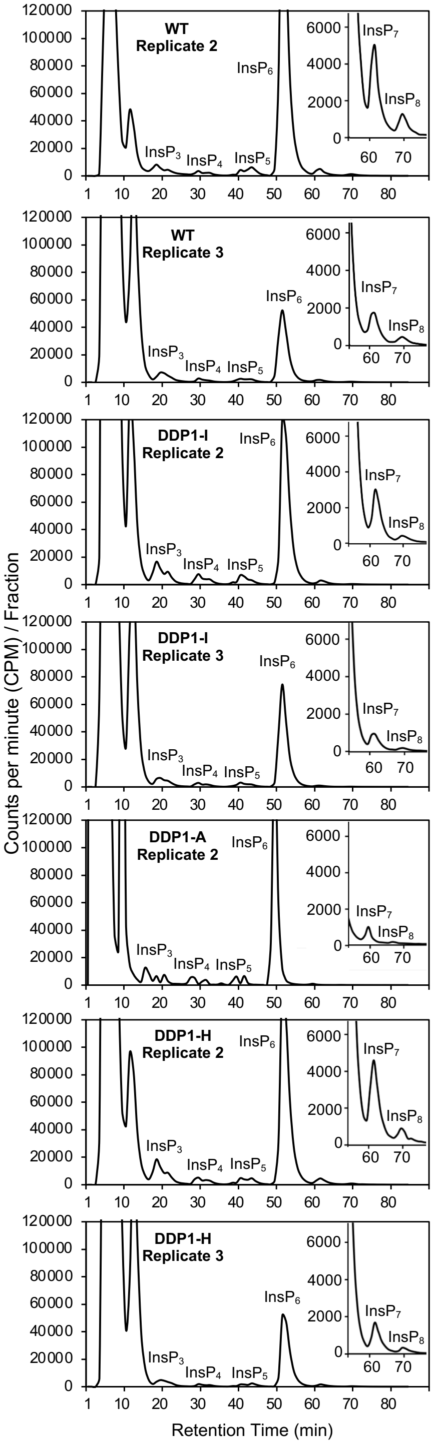

(b)

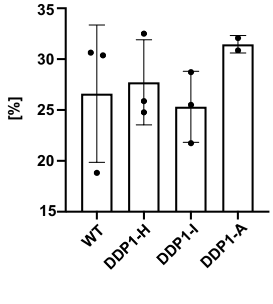

Supplementary Figure S1. Additional InsP and PP-InsP profiles of DDP1 OX transgenics. (a) WT and DDP1 OX transgenics were grown for 14 days on semi-solid 0.5X MS media with 0.2% agar then 100 µCi [^{3}H]-*myo-* inositol was added for 4 days. All InsPs were extracted and separated using anion exchange HPLC. (b) $InsP_8/InsP_7$ ratios in DDP1 OX transgenics. Error bars, SD of n = 2-3 independent experiments.

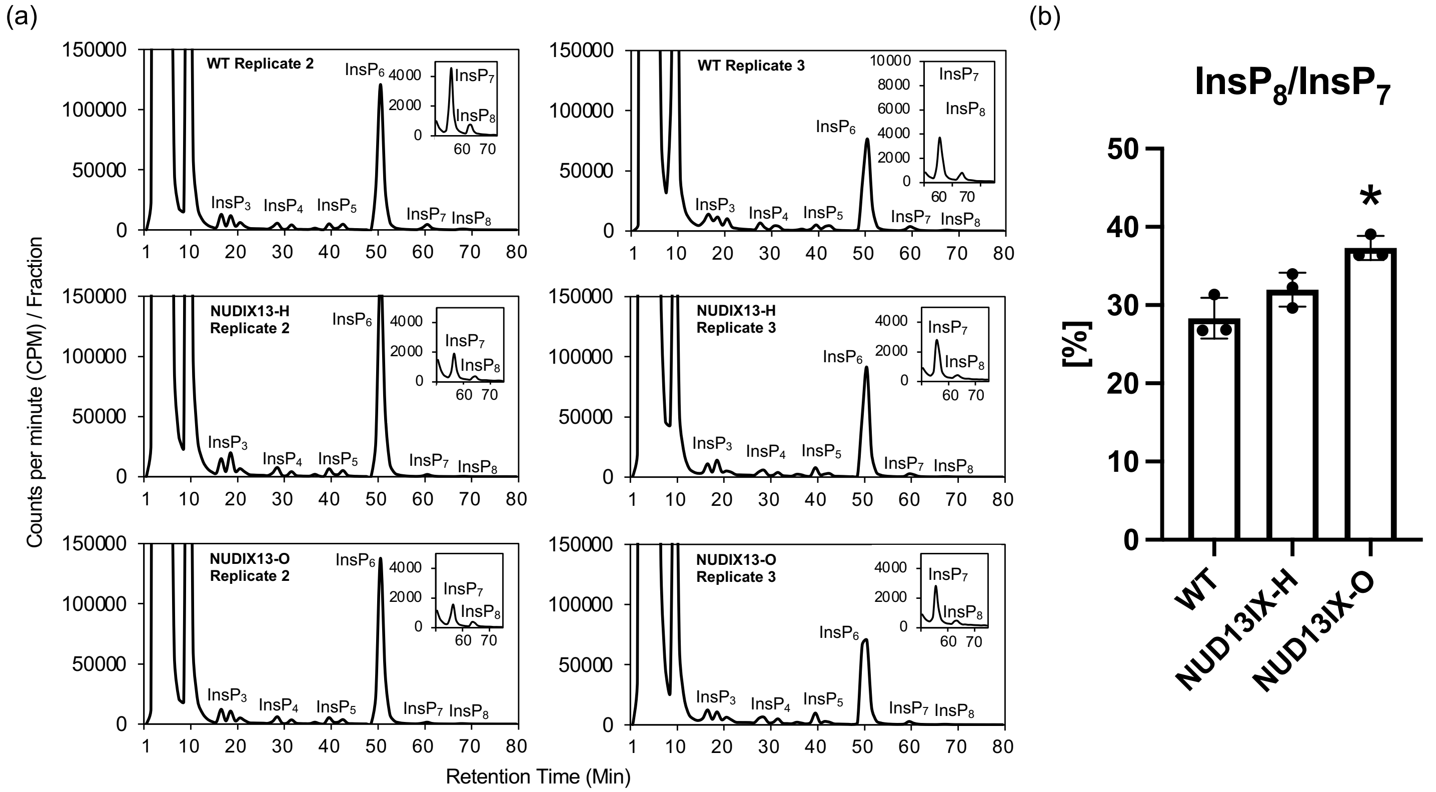

Supplementary Figure S2. Additional InsP and PP-InsP profiles of NUDIX13 OX transgenics. (a) WT and NUDIX13 OX transgenics were grown for 14 days on semi-solid 0.5X MS media with 0.2% agar then 100 µCi $[^3H]$-*myo*- inositol was added for 4 days. All InsPs were extracted and separated using anion exchange HPLC. (b) $InsP_8/InsP_7$ ratios in NUDIX13 OX transgenics. Asterisks show significant differences from WT; analyzed using Student's *t*-test; * $P < 0.05$, error bars show SD of $n = 3$.

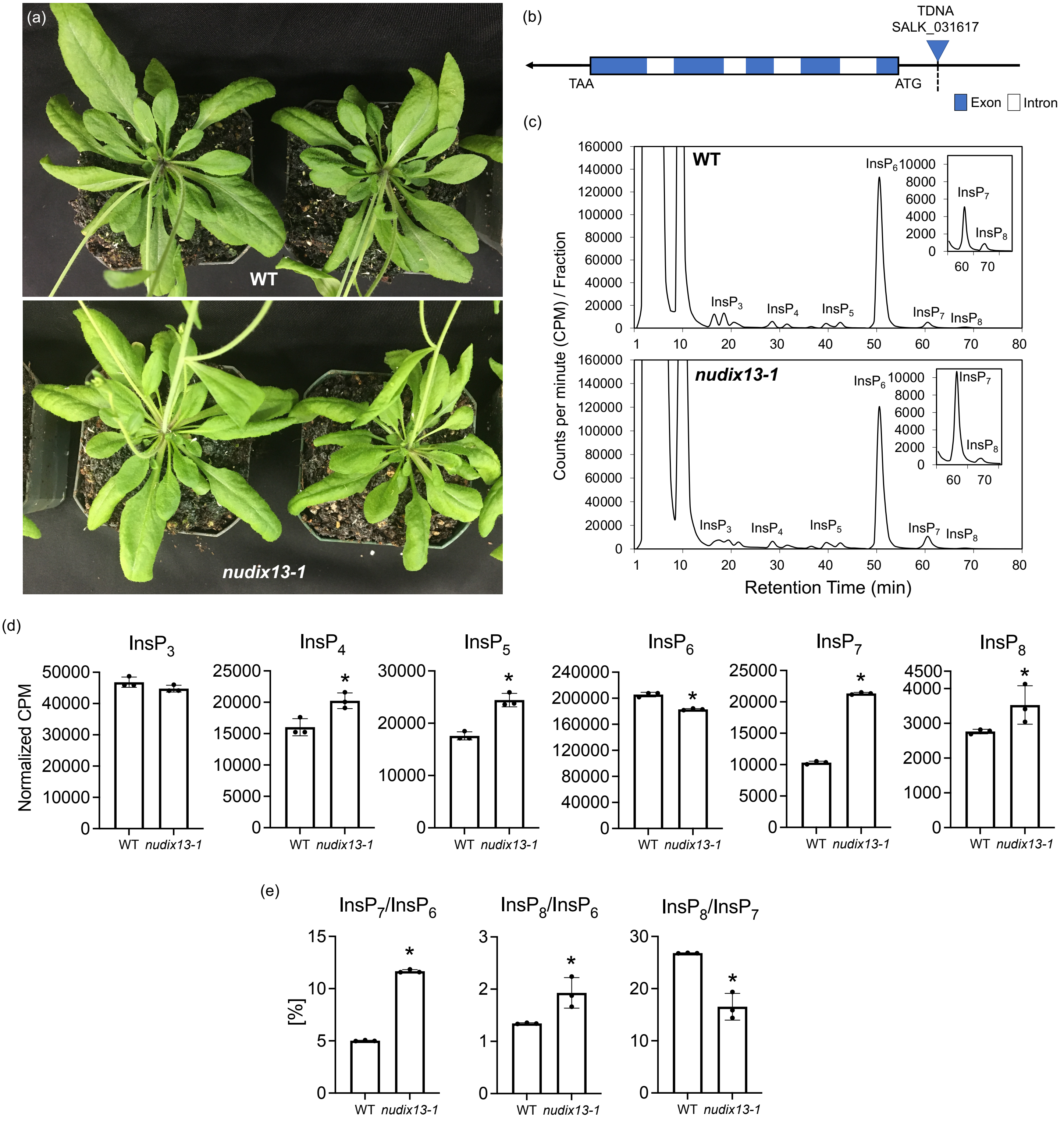

Supplementary Figure S3. InsP and PP-InsP profiles of *nudix13-1* plants. (a) 4-week-old *Arabidopsis* rosettes. (b) *NUDIX13* gene diagram showing exons (blue), introns (white), and promoter/untranslated regions (black lines). The T-DNA insertion is present in the promoter. (c) InsP profiles of WT and *nudix13-1* plants, representative of n = 3 independent replicates per genotype. Quantification of $InsP_{3\text{-}8}$ levels (d) and PP-InsP ratios (e) in both genotypes, analyzed using Student's *t*-test; * $P < 0.05$, error bars show SD of n = 3.

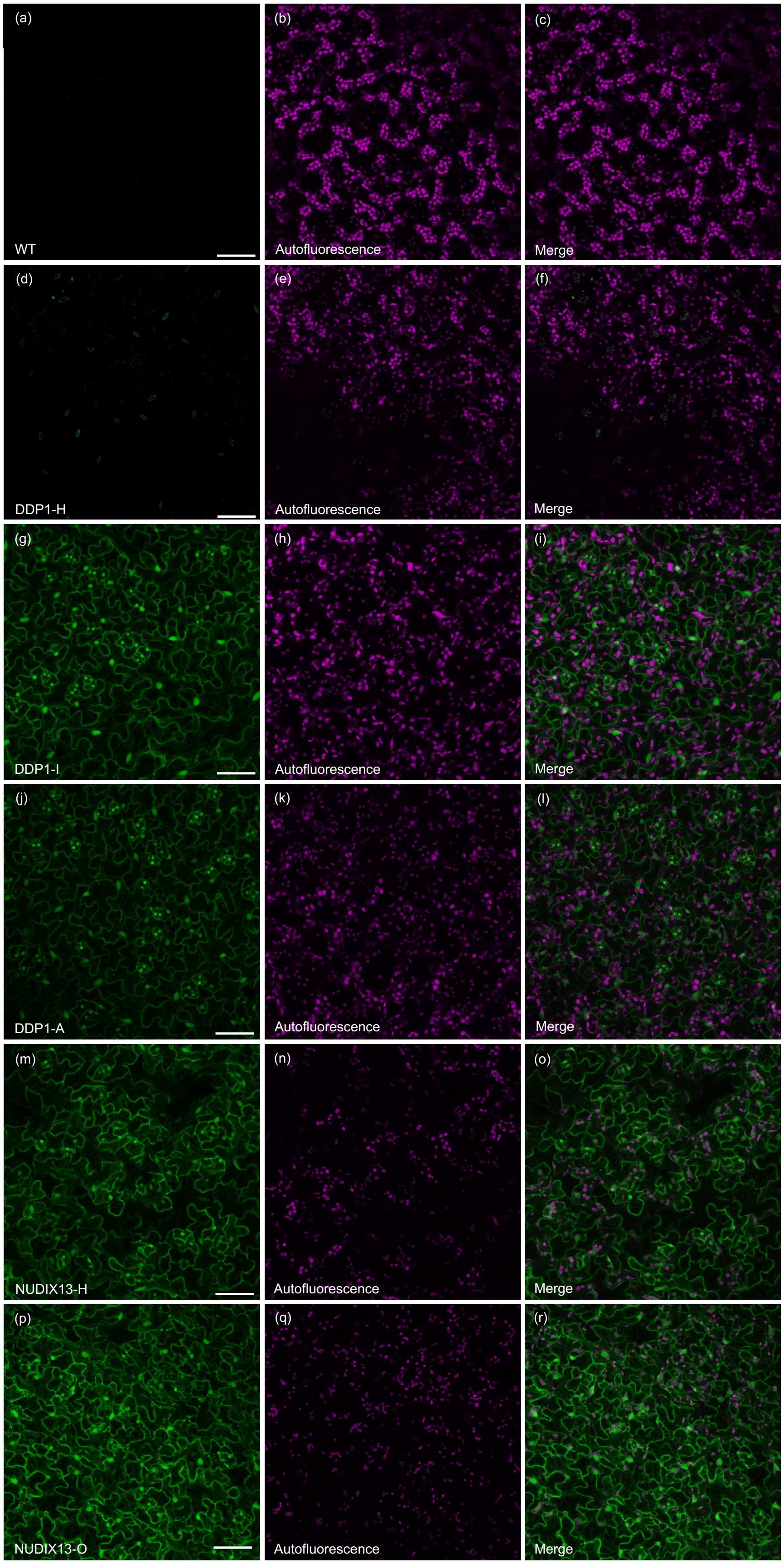

Supplementary Figure S4. DDP1-GFP and NUDIX13-GFP localization in *Arabidopsis* epidermal cells. Mature leaves of 3-week-old soil grown *Arabidopsis* were imaged. WT (a-c), DDP1-H (d-f), DDP1-I (g-i), DDP1-A (j-l), NUDIX13-H (m-o), and NUDIX13-O (p-r). Images (a, d, g, j, m, p) were imaged using the GFP channel and chlorophyll autofluorescence was imaged in images (b, e, h, k, n, q). Data in g, j, m, p is the same as presented Figures 6a, d-f. All images are presented as maximum intensity projections from confocal Z-stack optical sections. All scale bars = 50 μm.

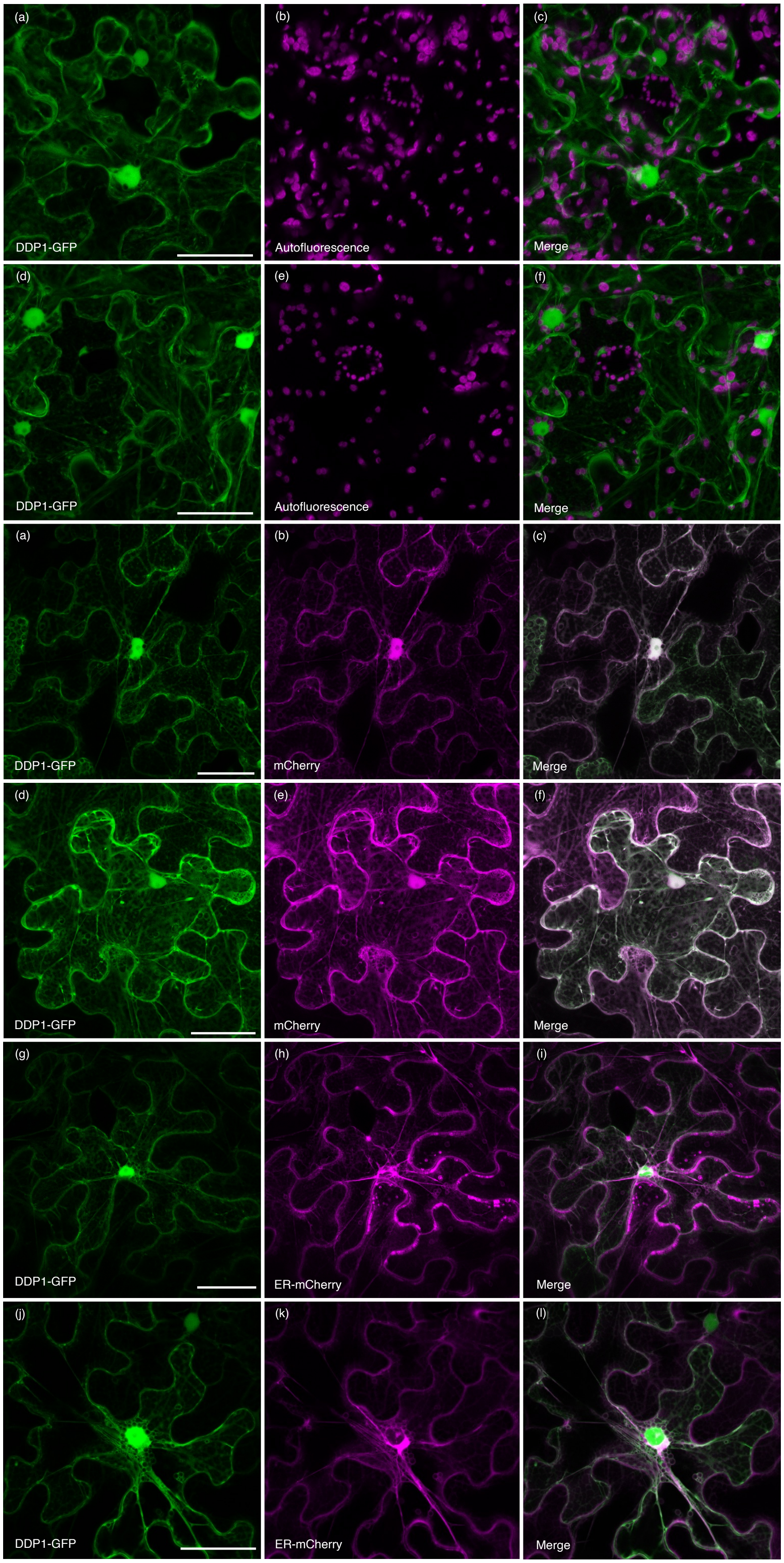

Supplementary Figure S5. Transient DDP1-GFP expression after 24 and 48 hours. DDP1-GFP was transiently expressed in *N. benthamiana* leaves and imaged 24 (a-c) and 48 (d-l) hours post-infiltration using confocal microscopy. Scale bar = 50 μm. Images a and d were imaged using the GFP channel and chlorophyll autofluorescence was imaged in b and e. DDP1-GFP (g,j,m,p) was co-expressed with unconjugated mCherry (h,k) and ER-mCherry (n,q). (i,l) show the DDP1-GFP + unconjugated mCherry merge and (o,r) show the DDP1-GFP + ER-mCherry merge. All images are presented as maximum intensity projections from confocal Z-stack optical sections. All scale bars = 50 μm.

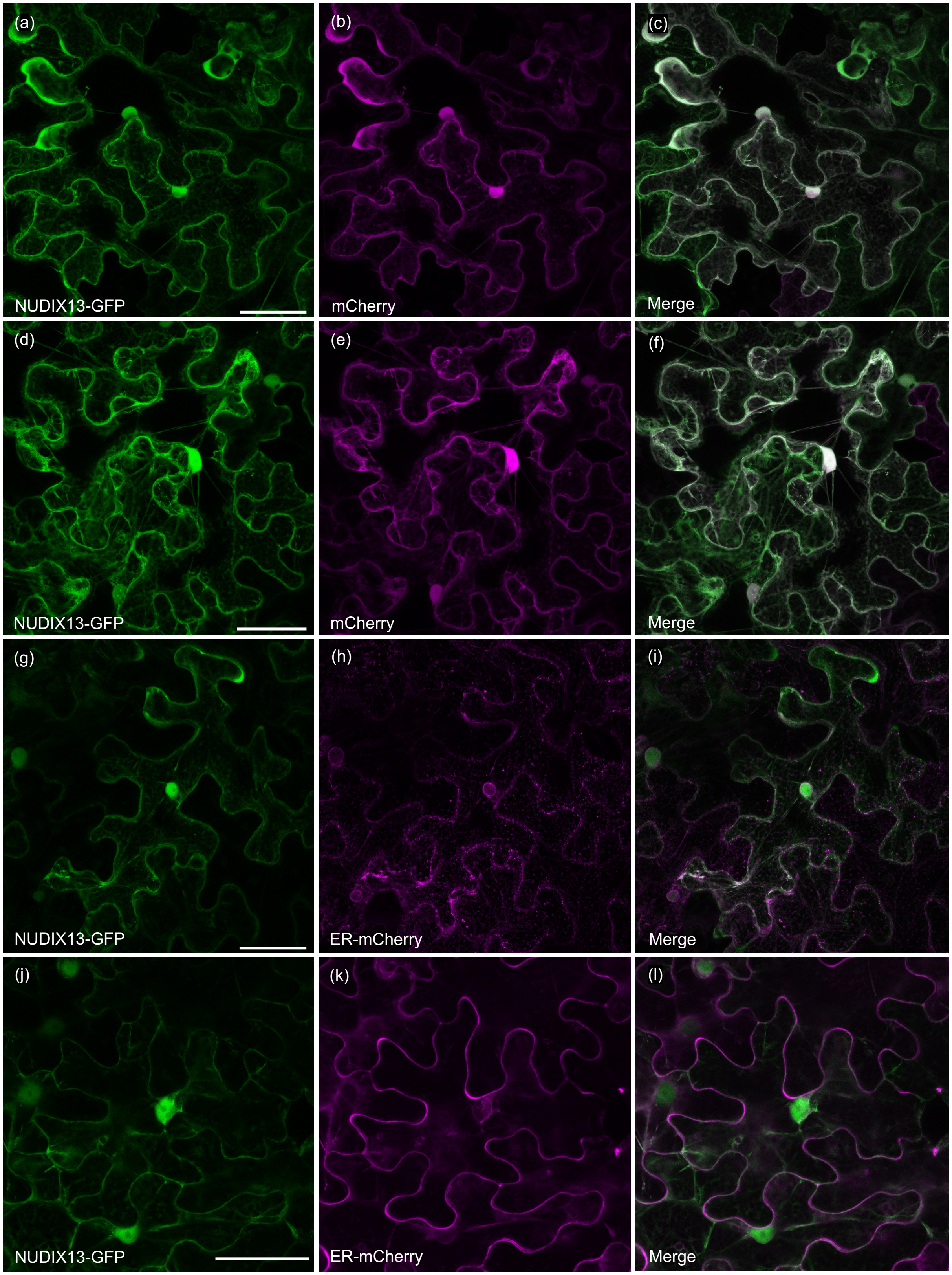

Supplementary Figure S6. Transient NUDIX13-GFP expression in *N. benthamiana* leaves 24- and 48-hours post-infiltration. NUDIX13-GFP (a,d,g,j) was co-expressed with unconjugated mCherry (b,e) and ER-mCherry (h,k). (c,f) show the DDP1-GFP + unconjugated mCherry merge and (i,l) show the NUDIX13-GFP + ER-mCherry merge. All images are presented as maximum intensity projections from confocal Z-stack optical sections. All scale bars = 50 μm.

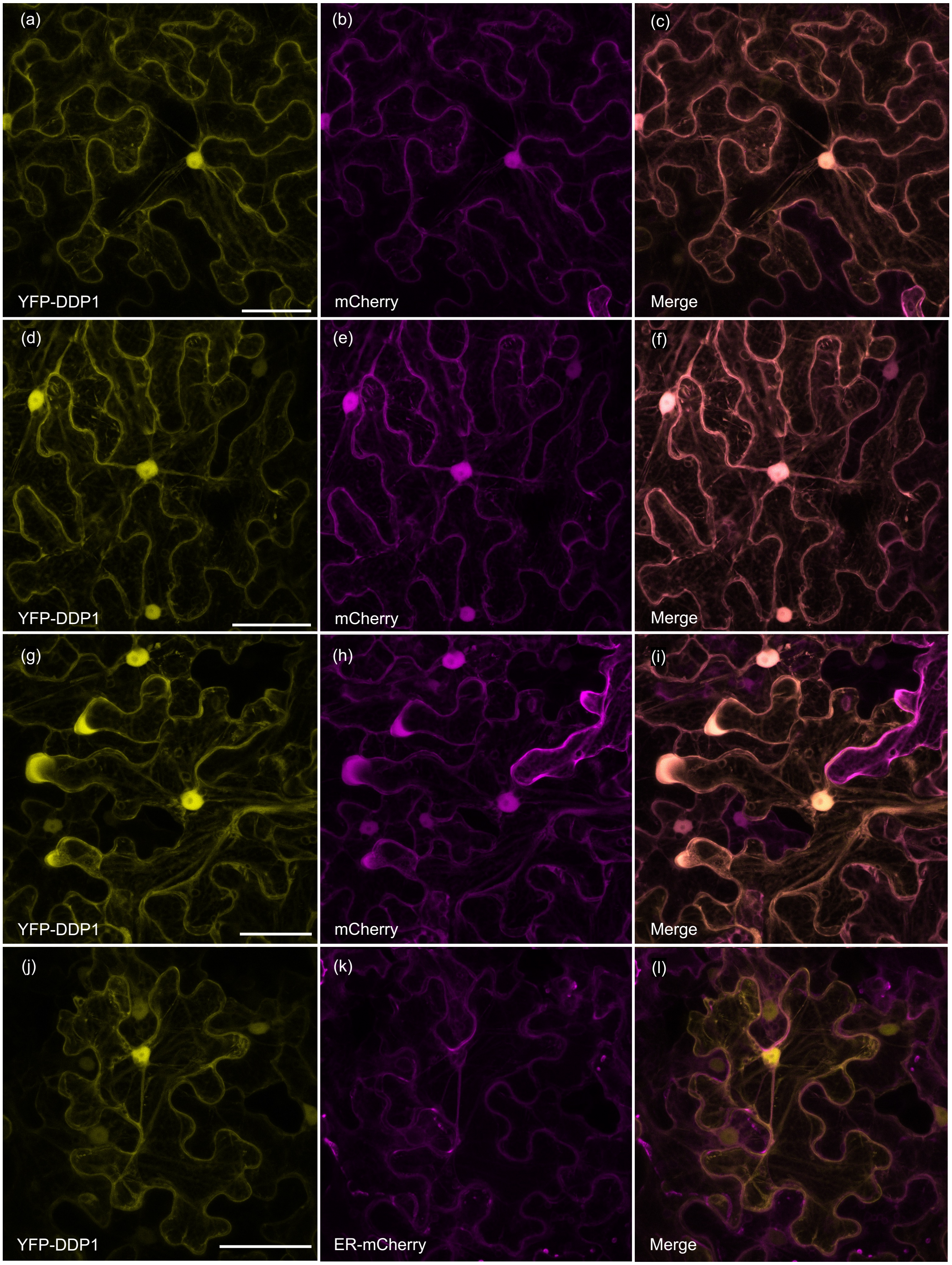

Supplementary Figure S7. ***N. benthamiana*** **leaves co-infiltrated with YFP-DDP1, unconjugated mCherry, and ER-mCherry.** Cells were imaged at 24 (a-c), 48 (d-f, j-l), and 72 (g-i) hours post-infiltration using confocal microscopy. (c,f,j) show the YFP-DDP1 + unconjugated mCherry merge and (l) shows the YFP-DDP1 + ER-mCherry merge. All images are presented as maximum intensity projections from confocal Z-stack optical sections. All scale bars = 50 μm.

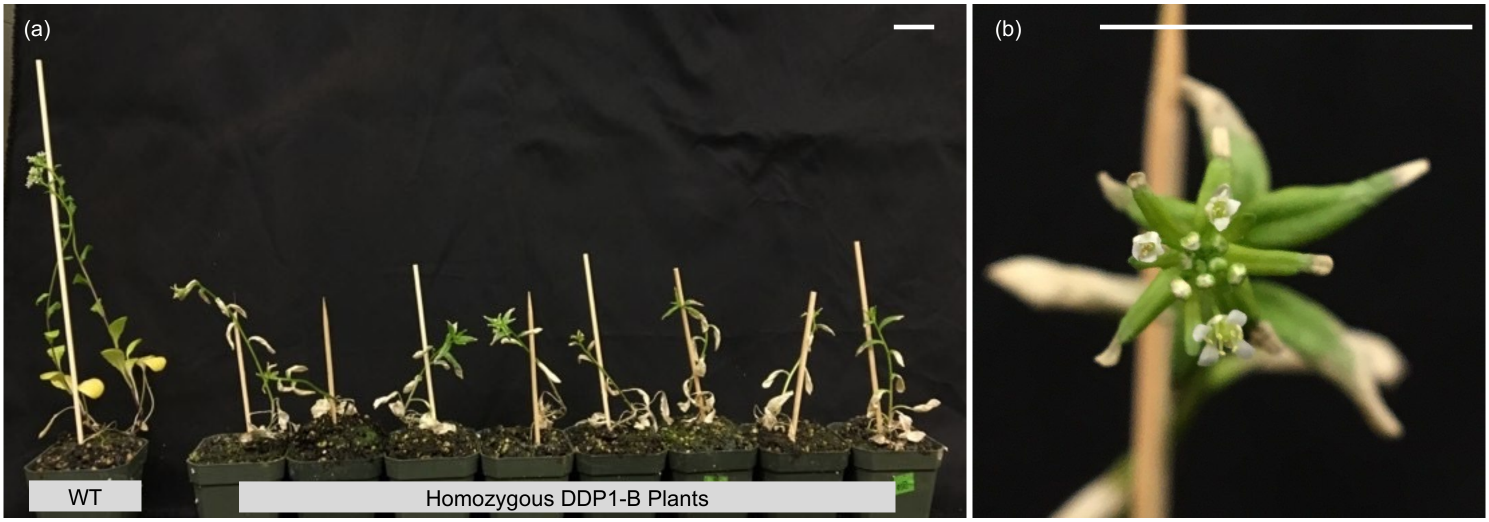

Supplementary Figure S8. Homozygous pennycress DDP1-B OX plants. Homozygous DDP1 overexpression severely stunted plant growth (a) and caused leaves to manifest detrimental amounts of leaf tip necrosis and chlorosis (b). (a-b) Scale bar = 1 inch.
