# Supplementary material for: Using native and synthetic genes to disrupt inositol pyrophosphates and phosphate accumulation in plants
Source: Plant Physiol. 2024 Oct 30;197(1):kiae582. doi: 10.1093/plphys/kiae582 (PMC11663554; doi:10.1093/plphys/kiae582)
Supplement: kiae582_Supplementary_Data [file kiae582_supplementary_data.zip › Table_S1_withReferences.pdf]

Supplementary Table S1. Oligonucleotide primers used in this study. PCR efficiencies for each primer pair shows the average of n = 3 technical replicates.

Gene name	Locus	Primer	Sequence (5'-> 3')	PCR Efficiency	cDNA Product (bp)	Product (gDNA)	Reference
PEX4 (Reference)	At5g25760	Forward	CTTAACTGCGACTCAGGGAATCTTCTAAG	100%	105	none	(Alford et al., 2012)
		Reverse	TCATCCTTTCTTAGGCATAGCGGC				
PS2	AT1G73010	Forward	TCGAGGAGATTAGATTGGAGGAAG	98%	142	none	(Land et al., 2021)
		Reverse	GACTGAGACACACGAAGAGCA				
PHT1;4	At2g38940	Forward	GAACGGTCCCAATAGTTTAGGTGAT	95%	78	none	(Nagarajan et al., 2011)
		Reverse	GAGTTGCTAGAGACAAGGAGAAAGAAA				
SPX1	At5g20150	Forward	GATTCCATTGTTGGAGCAAGA	100%	96	none	(Liu et al., 2011)
		Reverse	AATCTGTTAGCTTCTTCTATTGTA				
miR399d	At2g34202	Forward	GGTTGGATTACTGGGCGAATACT	87%	80	same as cDNA	(Bari et al., 2006)
		Reverse	CTCCTTTGGCAGAGAAGCATTTT				
IPS1	At3g09922	Forward	GGGATGGCCTAAATACAAAATGAA	100%	80	same as cDNA	(Martín et al., 2000)
		Reverse	TCCATATCTTAAAACGCTTTCCTTACA				
PHO1;H1	At1g68740	Forward	TACCGATTGGAGAATGAGCATCTAA	98%	96	none	(Stefanovic et al., 2007)
		Reverse	TTAGTCTTCTTCATCCACTTCTCTGAAAG				

Gene Name	Organism	Primer	Sequence (5' -> 3')	Product Size
DDP1 (YOR136w)	*S. cerevisiae*	Forward	CACCATGGGCAAAACCGCGGATAAT	560 bp
		Reverse	TTTGTCGTCTTTAATGATAGCAGACCT	
NUDIX13 (YOR136w)	*A. thaliana*	Forward	CACCATGTCGAATCTTTCTGCAAGAACA	609 bp
		Reverse	TTAGACTACAAAGCAGTAGCGAGG	

REFERENCES

Alford SR, Rangarajan P, Williams P, Gillaspy GE (2012) myo-Inositol Oxygenase is Required for Responses to Low Energy Conditions in Arabidopsis thaliana. Front Plant Sci **3**: 69

Bari R, Datt Pant B, Stitt M, Scheible W-R (2006) PHO2, MicroRNA399, and PHR1 Define a Phosphate-Signaling Pathway in Plants. Plant Physiol **141**: 988–999

Land ES, Cridland CA, Craige B, Dye A, Hildreth SB, Helm RF, Gillaspy GE, Perera IY (2021) A Role for Inositol Pyrophosphates in the Metabolic Adaptations to Low Phosphate in Arabidopsis. Metabolites **11**: 601

Liu T-Y, Aung K, Tseng C-Y, Chang T-Y, Chen Y-S, Chiou T-J (2011) Vacuolar Ca2+/H+ transport activity is required for systemic phosphate homeostasis involving shoot-to-root signaling in Arabidopsis. Plant Physiol **156**: 1176–1189

Martín AC, del Pozo JC, Iglesias J, Rubio V, Solano R, de La Peña A, Leyva A, Paz-Ares J (2000) Influence of cytokinins on the expression of phosphate starvation responsive genes in Arabidopsis. Plant J **24**: 559–567

Nagarajan VK, Jain A, Poling MD, Lewis AJ, Raghothama KG, Smith AP (2011) Arabidopsis Pht1;5 mobilizes phosphate between source and sink organs and influences the interaction between phosphate homeostasis and ethylene signaling. Plant Physiol **156**: 1149–1163

Stefanovic A, Ribot C, Rouached H, Wang Y, Chong J, Belbahri L, Delessert S, Poirier Y (2007) Members of the PHO1 gene family show limited functional redundancy in phosphate transfer to the shoot, and are regulated by phosphate deficiency via distinct pathways. Plant J **50**: 982–994
