# Supplementary material for: Using native and synthetic genes to disrupt inositol pyrophosphates and phosphate accumulation in plants
Source: Plant Physiol. 2024 Oct 30;197(1):kiae582. doi: 10.1093/plphys/kiae582 (PMC11663554; doi:10.1093/plphys/kiae582)
Supplement: kiae582_Supplementary_Data [file pp.02107.2024-s05.pdf › pp.02107.2024-s05.pdf]

Plant Physiology®

USING NATIVE PLANT AND SYNTHETIC GENES TO DISRUPT INOSITOL PYROPHOSPHATES AND PHOSPHATE ACCUMULATION IN PLANTS

Catherine Freed, Branch Craige, Janet Donahue, Caitlin Cridland, Sarah Williams, Chris Pereira, Jiwoo Kim, Hannah Blice, James Owen, and Glenda Gillaspy

Review Timeline:

Submission Date:	05-Jan-2024
Editorial Decision:	19-Feb-2024
Resubmission Received:	22-Jul-2024
Editorial Decision:	16-Sep-2024
Revision Received:	23-Sep-2024
Accepted:	28-Sep-2024

12-Feb-2024

Dr. Glenda E Gillaspy
University of Wisconsin-Madison College of Agricultural and Life Sciences
Madison, Wisconsin

RE: USING NATIVE PLANT AND SYNTHETIC GENES TO DISRUPT INOSITOL PYROPHOSPHATES AND PHOSPHATE ACCUMULATION IN PLANTS

Dear Dr. Gillaspy:

Thank you for giving us the opportunity to review your manuscript for publication in Plant Physiology. The reviewers found the manuscript of interest, but raised a number of concerns that will require your attention. As these concerns are of some gravity and will entail substantial changes to the manuscript including further experimental evidence, you are likely to need additional time to address them. I must therefore reject the manuscript in its present form. Nonetheless, if you are able to address the points raised in review, I would welcome your resubmission of a manuscript that incorporates your responses.

In particular, I direct your attention to the following comments for consideration in a resubmitted manuscript.

Both reviewers noted the importance - particularly in the agricultural context in which this manuscript is framed - of presenting data on the total amount of phosphate per plant, for both Arabidopsis and pennycress. The size reduction of the transgenics could cancel out the increased phosphate content, which is a key point that must be directly addressed.

Also relating to the agricultural context: The overwhelming global agricultural problem with phosphate is deficiency, not excess. The introduction and discussion should therefore be modified to better reflect this reality.

Each reviewer identified other - but different - major issues to address, most notably lack of enzyme kinetics and mutant data, with both of which I concur. As addressing both these issues would be an unreasonable amount of work, I would therefore ask you to address one or other of them. Either would entail adding experimental data to the manuscript.

Please note that any resubmission will be treated as a new entry to the system and is likely to go out for review.

If you have queries or concerns, you are welcome to contact me in the first instance.

Thank you again for allowing Plant Physiology to review your work. We look forward to hearing from you soon.

Sincerely,

Editor, Plant Physiology

-- Reviewer comments:
Reviewer #1 (Comments for the Author):

The manuscript by Freed et al reports overexpression phenotypes for yeast and plant NUDIX-type inositol pyrophosphatases in Arabidopsis and in Thlaspi arvense. Overall, I found this a well written manuscript that further supports previously reported molecular links between inositol pyrophosphate signaling molecules and phosphate homeostasis in plants. I summarize my comments and suggestions below:

1. Minor: Last paragraph of the introduction. It would be worth mentioning the InsP bound SPX-PHR complex structures in addition to Ried and Wild et al. https://pubmed.ncbi.nlm.nih.gov/35332155/ and Zhou et al., compare figure legend 1.

2. Minor: Given the recent identification of new PP-InsP isomers in plants, the authors may consider including isomer nomenclature in Figure 1.

3. Minor: Figure 3c. The authors may consider presenting these discrete data as scatter plot, not as a line graph.

4. Major: Figure 2. For the plants grown in panel (a) and in panel (e) the total Pi content should be reported.

5. Major: Table 1 / Figure 3 / Table 2 / Figure 4. Is it possible to estimate cellular concentrations (or total amount per tissue mass used) for InsP6, InsP7 and InsP8 instead of reporting % values? Did the authors consider determining which InsP7 and InsP8 isomers are affected in the DDP1ox lines? (https://pubmed.ncbi.nlm.nih.gov/33247133/)

6. Minor: "though the InsP7/InsP6 ratio, while substantially lower compared to that of WT, was not statistically significant (Figure S1b)" The ratio cannot be substantially lower and statistically insignificant at the same time, consider rephrasing.

7. Major: Paragraph "NUDIX13 hydrolyzes PP-InsPs and other predicted substrates in vitro" and associated Figure 5. It would be advisable to determine kinetic parameters and rate constants in order to compare the different substrates. Also, the PP-InsP isoform selectivity of DDP1 and NUDT13 should at least be considered and discussed.

8. Major: Based on the plasmid indicated in the methods section, it seems that the subcellular localisation studies of the DDP1 and NUDIX13 shown in Figure 6 rely on constructs driven by the 35S promoter, and not be the native promoter (in the case of NUDIX13). This should at least be clearly stated in the figure legend and associated text.

9. Minor: A comparison to this study https://pubmed.ncbi.nlm.nih.gov/35640071/ could be added to the discussion section.

Reviewer #2 (Comments for the Author):

This manuscript aimed to improve phosphorus (P) accumulation by regulation of inositol pyrophosphate as a signal of P nutrition in plants. The authors investigated the effect of overexpression of DDP1, NUDIX13 on Pi concentration in leaves. As expected, the overexpression lines showed higher Pi, although plant growth was lower than WT. The authors concluded that this result showed a potential to increase Pi accumulation. However, I did not agree the conclusion, because,
(1) growth reduction of overexpression lines was critical, then it was not clear whether phosphate uptake per plant was improved by this strategy.
(2) Total P and substantial plant biomass were not shown in this study, therefore total P uptake could not be estimated.
In terms of the agronomy, excess amount of P cultivated lands is not critical on crop production. If it is required to reduce P content from certain soils, cultivation of some crops having big biomass is reasonable. The groups Arabidopsis and Thlaspi unfortunately have small biomass, therefore it is clearly estimated that the increase of P accumulation is limited, if the effect of the genetic modification on P accumulation was proved.

On the other hand, I felt that the results shown in this study contained meaningful data, especially the results on the function of NUDIX13. Unfortunately, there were no results on loss-of-function by using mutants. Discussion about the homologous genes in Arabidopsis is also needed. Although some additional experiments were required such as mutant phenotyping, estimation of total P accumulation, and effects of NUDIX13 on Pi homeostasis (not only the concentration in whole leaf), I believe that the revised manuscript focusing on the function of the enzyme would be valuable.

Minor comments;
Materials and methods: the unit indicating light intensity should be improved. The word "photon flux density" is better, and the unit is generally "$\mu mol\ m^{-2}\ s^{-1}$".
"x *g*" should be used as the unit instead of "rpm".
"Tris-Cl" seems better to be indicated as "Tris-HCl".
Reference list should be uniformed, see instruction for authors.

Freed et al., Response to Review

1. One major issue we have addressed is expanding the scope of the work on NUDIX13 function, in terms of providing either enzyme kinetics or mutant data. We provide more compelling evidence that the protein encoded by *NUDIX13* is indeed one of the plant native enzymes that can break down inositol pyrophosphates. In this revised manuscript, we include data on a *nudix13* loss-of-function mutant (*nudix13-1*) which has elevated levels of inositol pyrophosphates, which is shown in Figure S3 and Table 2, and is discussed on pages 15-16, & 24. This data, along with the reported changes in NUDIX13 overexpressors (decreased in $InsP_7$ and $InsP_8$) and NUDIX13 enzyme assay data (hydrolysis of $InsP_7$ and $InsP_8$ *in vitro*), provide robust evidence supporting our conclusions on NUDIX13 gene function. We feel this new data complements the translational aspects of both DDP1 and NUDIX13 overexpression, which were our original focus.

2. We have also added data that addresses the amount of total phosphorus accumulated by individual DDP1 OX transgenics. Both reviewers noted that the size reduction of the DDP1 transgenics could cancel out the increased phosphorus content within the plant compared to WT plants. While we view our simple overexpression of DDP1 as a first step in the overall engineering of plants that can remediate phosphorus from the environment, we understand the importance of determining total phosphorus per individual plant. To do this, our collaborators at the USDA assisted us in developing a hydroponic system in which we could compare total P accumulation from individual plants. We note that this is a different system that necessitated much pilot and experimental work as we wanted to carefully control the amount of nutrients and growth conditions. Our new version of Figure 3 includes data in which we see that although DDP1-I shoot tissue accumulates a significantly higher percentage of P per gram compared to WT shoots (Figure 3e), WT plants accumulate significantly higher levels of P on an individual plant basis compared to DDP1-I OX plants (Figure 3f). However, we also note that WT plants of similar size to DDP1-I OX plants have significantly lower levels of P accumulation compared to DDP1-I plants (Figure 3g). This last point is important and should not be overlooked as future strategies to use the DDP1 gene for remediation could reduce the growth trade-off impacts. We have discussed this new and important data, and implications, on pages 10-12, 26-27.

3. We have addressed the editor and reviewers' point that the overwhelming global agricultural problem with phosphate is deficiency, not excess. We have included this point in a modified version of our introduction (page 2) and discussion sections (page 26).

We also appreciate the other comments and suggestions made by the reviewers to strengthen our manuscript. We address these points below:

Reviewer 1:

1. Minor: Last paragraph of the introduction. It would be worth mentioning the InsP bound SPX-PHR complex structures in addition to Reid and Wild et al. https://pubmed.ncbi.nlm.nih.gov/35332155/ and Zhou et al., compare figure legend 1.
Our response: We included the InsP bound SPX-PHR complex structures as described by Guan et al in the text on page 3.

2. Minor: Given the recent identification of new PP-InsP isomers in plants, the authors may consider including isomer nomenclature in Figure 1. ***Our response:*** We carefully thought about

this important issue, as the newly characterized $InsP_7$ isomers are clearly important in plants. However, at this time we do not yet know how these isomers function. We have mentioned and cited the current work in the figure legend, so that the reader is aware of these new findings, which does not change the interpretation of data we report.

3. Minor: Figure 3c. The authors may consider presenting these discrete data as scatter plot, not as a line graph. ***Our response:*** While we had also originally considered presenting the P*i* accumulation data as a scatter plot instead of a line graph, we found that the visualization of the plant's unique trends to accumulate P*i* overtime is obscured on scatter plots. We feel the data is best presented as a line graph for this reason.

4. Major: Figure 2. For the plants grown in panel (a) and in panel (e) the total P*i* content should be reported. ***Our response:*** Figure 3d contains data reported as P*i* per mg of plant tissue for 3,4,5,6 and 7 weeks of the genotypes shown in Figure 2. This is the standard way investigators are reporting total P*i* in these types of experiments. If the reviewer is referring to P per plant, then we have addressed this in the new data in Figure 3e, f, and g, and have discussed these data in the beginning of this letter.

5. Major: Table 1 / Figure 3 / Table 2 / Figure 4. Is it possible to estimate cellular concentrations (or total amount per tissue mass used) for $InsP_6$, $InsP_7$, and $InsP_8$ instead of reporting % values? Did the authors consider determining which $InsP_7$, and $InsP_8$ isomers are affected in the DDP1ox lines? (https://pubmed.ncbi.nlm.nih.gov/33247133/). **Our response:** Our work uses radiolabeling with ^{3}H-myo-inositol so that we have great sensitivity to measure changes in most/all InsPs and PP-InsPs. Although this is an older, classical technique, it is still state of the art for these molecules. It is not possible to estimate cellular concentrations using this approach as it is unclear, in any organism, how to label all pools of InsPs and PP-InsPs to homogeneity. Specifically, investigators using this technique have speculated that not all pools of InsPs and PP-InsPs get labeled to the same degree. In plants, it seems likely that there is a signaling pool of $InsP_6$, and also a P*i* storage pool. All of these factors make it difficult to convert the amount of radioactivity found in a fraction to a cellular concentration or a total amount. Henning Jessen's group reported a new technique in 2020 that uses capillary electrophoresis coupled to electrospray ionization mass spectrometry (ESI-MS), that can separate closely related isomers of PP-InsPs. Our HPLC method cannot do this, but it does give us nice sensitivity, and importantly, good reproducibility. Henning's group is the only group using this newer method, and one factor is the expense of the instrumentation. By using the upstream capillary electrophoresis, Henning's group has identified new isomers of $InsP_7$. While we think this is fantastic work, we point out that the functions of these new isomers are not yet clearly identified. For us, trying to implement a new technique that requires expensive instrumentation that we do not have available, so that we can examine isomers that may or may not be important, seems like a minor sidetrack. We have already shown that overexpression of DDP1, for example, reduces the pool of $InsP_7$ and $InsP_8$. Teasing out the isomers seems like a separate project to us.

6. Minor: "though the $InsP_7/InsP_6$ ratio, while substantially lower compared to that of WT, was not statistically significant (Figure S1b)" The ratio cannot be substantially lower and statistically insignificant at the same time, consider rephrasing. **Our response:** We have made this change on page 9 as requested.

7. Major: Paragraph "NUDIX13 hydrolyzes PP-InsPs and other predicted substrates in vitro" and associated Figure 5. It would be advisable to determine kinetic parameters and rate constants in order to compare the different substrates. Also, the PP-InsP isoform selectivity of DDP1 and NUDT13 should at least be considered and discussed. **Our response:** Instead of focusing on kinetic parameters to expand the scope of our studies, we isolated and characterized a *nudix13-1* mutant, as suggested by the editor. We do believe eventual kinetic analyses and substrate specificity studies will be informative on NUDIX13 and other NUDIX enzymes from plants, but this is out of scope of our current work. We do feel that the scope of our current work is rather large given our focus on providing a proof of principle that overexpression of the yeast and plant phosphatases can turn on a phosphate uptake pathway. We have added this information about PP-InsP selectivity for DDP1 regarding PP-InsPs (page 5). We have also addressed the likely isomer specificity in DDP1 and NUDIX13 in the discussion (page 24).

8. Major: Based on the plasmid indicated in the methods section, it seems that the subcellular localization studies of the DDP1 and NUDIX13 shown in Figure 6 rely on constructs driven by the 35S promoter, and not be the native promoter (in the case of NUDIX13). This should at least be clearly stated in the figure legend and associated text. **Our response:** We did use the 35S promoter in our localization studies and have amended the text to indicate this (pages 18-19).

9. Minor: A comparison to this study https://pubmed.ncbi.nlm.nih.gov/35640071/ could be added to the discussion section. ***Our response:*** We added the Gaugler et al. work to the revised discussion on page 22 as requested.

Reviewer #2:

1. Materials and methods: the unit indicating light intensity should be improved. The word "photon flux density" is better, and the unit is generally "µmol m-2 s-1". ***Our response:*** We have replaced the units on page 27 as requested.

2. "x g" should be used as the unit instead of "rpm". ***Our response:*** We have made this change on pages 30-31.

3. "Tris-Cl" seems better to be indicated as "Tris-HCl". ***Our response:*** We made this change on pages 29-30 as requested.

4. Reference list should be uniformed, see instruction for authors. ***Our response:*** We have updated our reference list and standardized to the Plant Physiology style guide.

16-Sep-2024

Dr. Glenda E Gillaspy
University of Wisconsin-Madison College of Agricultural and Life Sciences
Madison, Wisconsin

RE: USING NATIVE PLANT AND SYNTHETIC GENES TO DISRUPT INOSITOL PYROPHOSPHATES AND PHOSPHATE ACCUMULATION IN PLANTS

Dear Dr. Glenda Gillaspy:

We are pleased to accept your manuscript for publication in the Plant Physiology. This acceptance is contingent on revision based on the comments of our reviewers. In particular, please consider the following:

First, I apologize that it took a bit longer than usual to go through the entire process. We have our expert reviews now. Both Reviewers greatly appreciate your effort to improve the manuscript.

Reviewer 2 points out remaining minor items, which all seem important and critical. Especially, Reviewer 2 identified some inconsistency on Table 2, and found some critical errors/typos in the Methods section. They also ask to re-plot the figure with specific contents (not concentration) of phosphate. Please revise the manuscript according to Reviewer 2's comments.

Please highlight all changes and include a detailed annotation to changes to the text, with line numbers, and noting your responses to the comments.

To submit your revised manuscript, click:

Link Not Available

If you cannot return the revised manuscript within 8 weeks of receipt, please let us know. Otherwise, we will assume that you have elected not to revise the manuscript and are withdrawing it.

Thank you for allowing us to review your work. We look forward to hearing from you soon.

Sincerely,

Editor, Plant Physiology

-- Reviewer comments:
Reviewer #1 (Comments for the Author):

I find the revised manuscript to be significantly improved. I had suggested to perform NUDT13 enzyme kinetics, but the analysis of the nudt13 loss-of-function mutant is actually the more informative experiments. I have no further comments.

Reviewer #2 (Comments for the Author):

The current manuscript is well revised, and I generally agree with the revised parts. However, further revisions are needed as below, especially in the explanation of additional experiments.

Fig.3e and L215: P concentration should be indicated by not percentage but substantial unit (e.g. mgP per g dry weight).

The data presented in Table 2 do not match the values in the context, which makes the data unreliable. In addition, it would be more appropriate to present the values in Table 2 in terms of CPM of InsPs, including WT, rather than relative values (%), and a discussion on a CPM basis would be necessary.

L536-537: Is pH 6.0{plus minus}5 is accurate? pH 6.0 {plus minus} "0.5" seems adequate.
L538: I do not understand what "from 1600 HR to 800 HR" means.

Table 2: The data of InsP8 for nudix13-1 was highlighted in green, regardless the value was less than 120%.

Often there is no space between data and units: e.g. L219: 200mg, L536: 479mg/L, 0.05mL, L601 and L608: 20mM.

My previous suggestion is not corrected:
"Tris-Cl" seems better to be indicated as "Tris-HCl".

L550: city and country name are required for Carl Zeiss.
L586: provider's name, city and state information are required for ICP.
L590: Invitrogen first appears in L589.
Fig.2g: text in the picture should be "DDP1-I" instead of DDP1-1.
Fig.3de: the unit information should be on dry (or fresh) weight basis.
Fig.3h: "miR399d" should not be indicated with a line break.

Freed et al., Response to Review

Thank you so much for your careful consideration and acceptance of our manuscript. Our team appreciates the time and effort taken to review these changes. We have adjusted the manuscript to include the minor revisions suggested by Reviewer 2. We address these points below:

Reviewer 2:
The current manuscript is well revised, and I generally agree with the revised parts. However, further revisions are needed as below, especially in the explanation of additional experiments.

1. Fig.3e and L215: P concentration should be indicated by not percentage but substantial unit (e.g. mgP per g dry weight). ***Our response:*** We have adjusted the graph to include mg P/g dry mass in figure 3 and on lines 215-216.

2. The data presented in Table 2 do not match the values in the context, which makes the data unreliable. In addition, it would be more appropriate to present the values in Table 2 in terms of CPM of InsPs, including WT, rather than relative values (%), and a discussion on a CPM basis would be necessary. ***Our response:*** Reviewer 2 astutely pointed out the discrepancies between the text and table 2, specifically with the nudix13-1 values not matching the text. We accidentally included an earlier version of the table and have now added the correct nudix13-1 values on revised Table 2 for all InsPs and PP-InsPs. We also corrected a mistake in line 268 that stated $InsP_3$ and $InsP_4$ levels were significantly higher in *nudix13-1* however, this was meant to be $InsP_4$ and $InsP_5$ given the data in Figure S3. This fix also fixes the inconsistency between the text, Table 2, and Figure S3.

Reviewer 2 also proposed the possibility of switching the average % of each InsP compared to WT in Table 2 to presenting these values in CPM. We considered this possibility when constructing the paper however, we decided that comparing the changes in InsPs from the total WT InsP pools as percentages adds great value as it allows readers, especially those newer to the field of PP-InsPs, to quickly compare changes between transgenic and mutant genotypes. Additionally, our data presentation is somewhat unique as we are presenting all of the InsP and PP-InsP species we can quantify using our method whereas other authors in the plant PP-InsP field typically only present ratios, traces, and/or CPM of the higher InsPs ($InsP_6$, $InsP_7$, and $InsP_8$). Given this, we have decided to keep table 2 to show the percent of the total WT InsP and PP-InsP pools rather than switch to total CPMs.

3. L536-537: Is pH 6.0{plus minus}5 is accurate? pH 6.0 {plus minus} "0.5" seems adequate.
Our response: We have adjusted this error and changed the "5" to "0.5" on line 537.

4. L538: I do not understand what "from 1600 HR to 800 HR" means.
Our response: We have adjusted this sentence to make the time of day that the pump was off clearer on line 538.

5. Table 2: The data of $InsP_8$ for *nudix13-1* was highlighted in green, regardless the value was less than 120%. ***Our response:*** Given the addition of the revised Table 2, this comment is no longer an issue and the $InsP_8$ value is within the threshold.

6. Often there is no space between data and units: e.g. L219: 200mg, L536: 479mg/L, 0.05mL, L601 and L608: 20mM.
Our response: We have fixed all instances of this throughout the manuscript. Spaces between the data and units have been added to the following lines: 219 (200 mg), 536-537 (479 mg/L, 0.05 mL/sec), 603 (20 mM), 608 (20 mM), 610 (20 mM), 611 (5 mM), 626 (1 X), 630 (1 X), 633 (5 mM), and 638 (1 mM).

7. My previous suggestion is not corrected: "Tris-Cl" seems better to be indicated as "Tris-HCl".
Our response: We have fixed all instances of this. The adjustments made on the following lines: 603, 608, 610, 632, 638, 641, & 646.

8. L550: city and country name are required for Carl Zeiss.
Our response: We have added the city, state, and country name on line 550.

9. L586: provider's name, city and state information are required for ICP.
Our response: We have added the city, state, and country name for Brookside Labs, the ICP and MARS microwave on lines 585-588.

10. L590: Invitrogen first appears in L589.
Our response: We have adjusted lines 590-591 by moving the company name, city, state, and country name earlier in the text on line 590.

11. Fig.2g: text in the picture should be "DDP1-I" instead of DDP1-1.
Our response: We have fixed this error and uploaded the updated file for Figure 2.

12. Fig.3de: the unit information should be on dry (or fresh) weight basis.
Our response: We have changed the graph to present data as mg P/g dry mass and have uploaded the updated file for Figure 3.

13. Fig.3h: "miR399d" should not be indicated with a line break.
Our response: We have fixed this error and uploaded the updated file for Figure 3.

28-Sep-2024
Dr. Glenda E Gillaspy
University of Wisconsin-Madison College of Agricultural and Life Sciences
Madison, Wisconsin

MSID: PP2024-RA-02107DR1
MS TITLE: USING NATIVE PLANT AND SYNTHETIC GENES TO DISRUPT INOSITOL PYROPHOSPHATES AND PHOSPHATE ACCUMULATION IN PLANTS

Dear Dr. Glenda Gillaspy:

I am pleased to inform you that your revised manuscript "USING NATIVE PLANT AND SYNTHETIC GENES TO DISRUPT INOSITOL PYROPHOSPHATES AND PHOSPHATE ACCUMULATION IN PLANTS" has been accepted for publication in Plant Physiology. Your manuscript will be checked for consistency with journal formatting and image standards by one of our Science Editors, who will contact you shortly. They may have a number of queries and suggestions for you. Please keep in mind that they work to increase the online visibility of your article and its attraction for readers from the widest of backgrounds. You will have the opportunity to review any revisions, and I hope you will work constructively with them to get the most out of your research. You will also have the opportunity to review all changes when you receive the author proofs for your article.

Once validated for publication, your article will be sent to a Science Editor for a final review of your manuscript and source files. After your final files are uploaded, your article will appear in the next available issue and the online Preview version of your article will be posted after your signed Copyright and Checklist forms and final source files are received.

Please be sure to include the following in your manuscript file:
Harvard style citations
List of all author contributions and funding information at the beginning of manuscript after the titles and author list.
List all supplemental materials associated with the article, with titles, at the end of the ms, before the references and acknowledgments section.
Complete Figure Legends for all figures appearing in the article listed before the Literature Cited.

LICENSE INFORMATION Shortly after your paper is sent to production, you will receive an email from our publisher's author support team, **SciPris**, with information on article processing charges, as well as the types of Open Access license you would be able to purchase. The accepted version of your manuscript will not appear on our Advance Articles page until the license is selected and signed, so please look out for an email from SciPris. For more information see the Article Publication Fees section of our author guidelines: https://academic.oup.com/plcell/pages/General-Instructions

Finally, we encourage your submission of artwork for the journal cover. If there is an image or illustration related to your paper that you would like to have considered, please email it in .pdf or .tif format to clowe@aspb.org. An image title, brief, 2-3 sentence description of the image, and the name of the person credited for the image are also encouraged. If your image is selected to appear on the online cover of the journal, you will be contacted with the scheduled issue date and further information.

Thank you and congratulations on your Plant Physiology paper!

Sincerely,
Editor, Plant Physiology

=====
FIRST AUTHOR PROFILES
=====

We recognize the hard work that goes into being First Author of a Plant Physiology paper by publishing a "First Author Feature" for each First Author alongside your paper.

If the first author(s) would like to submit information for a feature on Facebook and Twitter, fill out the form by clicking on the link below:

Plant Physiology First Author Feature Form: https://forms.office.com/r/ZBCGZfhm8F
If you cannot access the form through the link above, please contact Rachel at rbelsky@aspb.org.

To upload an image of yourself to accompany your profile, please put your photo in the correct journal folder at the link below.
You must label your image: Author First Name Last Name Article Title.

First Author Profile Image Dropbox: https://www.dropbox.com/scl/fo/xz029p7qjr2jml2rp2kmq/h?dl=0&rlkey=6fkxcaqmxx35ap152b2osy1fz

=====
IMPORTANT REMINDER: PEER REVIEW REPORTS
=====

If you opted to publish a peer review report along with your article during the original submission process, it will be prepared by the editorial staff and publicly posted with your manuscript, inside the zip file that contains any other supplemental material. As a reminder, the peer review report is a public record of all comments from editors and reviewers, as well as your prior responses, as you received them in the decision letters for each draft of your manuscript. If you agreed to publish this report and have changed your mind, or are not sure if you selected this option, please contact the editorial office as soon as possible before signing the license agreement from our publisher.

-- Reviewer comments:
